# Structural Insights into a Wildtype Domain of the Oncoprotein E6 and Its Interaction with a PDZ Domain

**DOI:** 10.1371/journal.pone.0062584

**Published:** 2013-04-30

**Authors:** André Mischo, Oliver Ohlenschläger, Peter Hortschansky, Ramadurai Ramachandran, Matthias Görlach

**Affiliations:** 1 Biomolecular NMR Spectroscopy, Leibniz Institute for Age Research - Fritz Lipmann Institute, Jena, Germany; 2 Molecular and Applied Microbiology, Leibniz Institute for Natural Product Research and Infection Biology - Hans-Knöll-Institute, Jena, Germany; Harvard Medical School, United States of America

## Abstract

The high-risk human papilloma virus (HPV) oncoproteins E6 and E7 interact with key cellular regulators and are etiological agents for tumorigenesis and tumor maintenance in cervical cancer and other malignant conditions. E6 induces degradation of the tumor suppressor p53, activates telomerase and deregulates cell polarity. Analysis of E6 derived from a number of high risk HPV finally yielded the first structure of a wild-type HPV E6 domain (PDB 2M3L) representing the second zinc-binding domain of HPV 51 E6 (termed 51Z2) determined by NMR spectroscopy. The 51Z2 structure provides clues about HPV-type specific structural differences between E6 proteins. The observed temperature sensitivity of the well-folded wild-type E6 domain implies a significant malleability of the oncoprotein *in vivo*. Hence, the structural differences between individual E6 and their malleability appear, together with HPV type-specific surface exposed side-chains, to provide the structural basis for the different interaction networks reported for individual E6 proteins. Furthermore, the interaction of 51Z2 with a PDZ domain of hDlg was analyzed. Human Dlg constitutes a prototypic representative of the large family of PDZ proteins regulating cell polarity, which are common targets of high-risk HPV E6. Nine C-terminal residues of 51Z2 interact with the second PDZ domain of hDlg2. Surface plasmon resonance in conjunction with the NMR spectroscopy derived complex structure (PDB 2M3M) indicate that E6 residues N-terminal to the canonical PDZ-BM of E6 significantly contribute to this interaction and increase affinity. The structure of the complex reveals how residues outside of the classical PDZ-BM enhance the affinity of E6 towards PDZ domains. Such mechanism facilitates successful competition of E6 with cellular PDZ-binding proteins and may apply to PDZ-binding proteins of other viruses as well.

## Introduction

Infections are responsible for 17.8% of all cancers [1]. Several human papilloma virus (HPV) types are the most frequent viral cancer-causing agents and are linked to well over half a million incidences of cervical cancer each year [1], [2], [3]. Though vaccines against HPV types 6, 11, 16 and 18 are available, these HPV types cover only ∼70% of cervical cancer cases and the vaccines do not cure already existing infections [3], [4]. Development of therapies against HPV-caused malignancies thus requires further mechanistic insight into how oncogenic HPV drive tumor development and maintenance.

The HPV oncoproteins E6 and E7 of tumor-associated so-called “high-risk” HPVs prevent the differentiation of HPV-infected keratinocytes and immortalize primary, cultured human keratinocytes [5], [6], [7]. Moreover, primary human cervical carcinoma cells require E6 and E7 expression for proliferation [8]. Upon depletion of E6 and/or E7, HPV-transformed cervical cancer derived cell lines undergo apoptosis or senescence [9], [10], [11], [12], [13], [14]. Apoptotic HPV positive cancer cells may transform human primary fibroblasts by horizontal gene transfer of the E6/E7 ORF [15]. The double-stranded DNA genome of HPV is replicated as an episome in infected cells, but almost 90% of cervical carcinomas contain genome-integrated HPV sequences, encompassing at least the E6/E7 open reading frame (ORF) [16]. The integration and subsequent loss of episomal HPV sequences leads to absence of E2 which usually negatively regulates the transcription of E6/E7 [16]. Collectively these findings suggest that the “malicious couple” E6 and E7 is necessary for formation and maintenance of cervical cancer.

The HPV oncoprotein E6 has no known enzymatic activity. It interacts with numerous cellular proteins and these interactions contribute to reprogramming keratinocytes so as to prevent normal differentiation and to maintain cellular replication competence in order to amplify the HPV genome [17], [18].

The inactivation of p53 is a general hallmark of tumorigenesis and tumors frequently show mutations and/or reduced p53 levels [19]. E6 interacts with p53 [20] and the E3 ubiquitin ligase E6AP [21]. The E6 interaction leads to an alteration of the substrate specificity of E6AP ultimately resulting in proteasomal p53 degradation [22], [23]. E6 also induces p53 degradation independent of E6AP [24] and also independent of ubiquitin-mediated proteasomal degradation [25]. Interestingly, p53 degradation is a common mode of action of the high-risk HPVs and is rarely found for other HPV types [26], [27].

A second common feature of tumors constitutes an increase in telomerase activity [19]. This increase results both from direct interaction of E6 with the enzyme and *via* induction of telomerase reverse transcriptase at the transcriptional level [28], [29]. Again, this property appears to be shared primarily among the oncogenic HPV types such as *e.g.* HPV 16, 18 and 51 [30].

Furthermore, E6 of high-risk types interacts with a number of PDZ-domain containing proteins (PDZ proteins) *via* its C-terminal PDZ-binding motif (PDZ-BM) [31], [32]. These PDZ proteins, usually containing multiple PDZ domains, act frequently as hub proteins in signaling and/or regulation of cell polarity and are typically degraded upon E6 interaction [31]. Deletion of the PDZ-BM from the high-risk type HPV 31 E6 results in a retarded growth-rate of infected cells and a reduced copy number of the viral episome [33]. The absence of the PDZ-BM of HPV 16 E6 in an *in vivo* mouse model resulted in smaller cervical tumors [34]. This strongly suggests a tumor promoting mechanism based upon E6’s targeting of PDZ proteins. Interestingly, PDZ proteins are common targets for human tumorigenic and nontumorigenic viruses [35], [36]. The proteins targeted by these viruses control formation of tight junctions, cell adhesion and apoptosis [36]. This targeting appears to be a common strategy to support viral replication and transmission to new hosts. A prominent case here constitutes the observation that one single mutation in the PDZ-BM of the envelope protein of rabies virus dramatically changed its PDZ protein target spectrum and resulted in a switch from the virulent to an attenuated state [37].

One PDZ domain containing protein targeted by E6 for degradation *in vivo* is the multi-domain protein hDlg (human Dlg/hDlg1/SAP-97) [38]. The E6 dependent reduction of hDlg levels has been demonstrated by an *in vitro* degradation assay for a number of high-risk HPVs, among them HPV 16, 18 and 51 [32]. Human Dlg is part of the Scribble polarity complex that controls basolateral polarity [39] and it is required for adherens junction formation and differentiation of epithelial cells [40]. Human Dlg is expressed in human keratinocytes and localizes to nuclear, cytoplasmic, membrane-associated and cytoskeletal pools that are thought to exert different functions [41]. Different isoforms of hDlg with different cellular localization and translation efficiency by alternative splicing [41], [42]. The hDlg-APC (Adenomatous Polyposis Coli) complex negatively regulates cell cycle progression from G0/G1 to S phase [43] and hDlg-depleted keratinocytes show an increased resistance to anoikis [44]. Human Dlg recruits the Src homology 3 domain-containing (RhoG-specific) guanine nucleotide exchange factor (SGEF) to the cytoskeleton and induces SGEF activity [45]. Interestingly, this cytoskeletal pool of hDlg is not degraded by E6 [45]. Moreover, E6 interacts with SGEF in an hDlg-dependent manner and maintains high RhoG activity, thereby increasing invasive capacity [45]. Thus, the role of hDlg in tumor formation seems to be ambivalent and E6 apparently specifically abrogates certain tumorsuppressive hDlg activities [41].

Full-length E6 consists of approximately 150 residues and includes two zinc-binding domains (ZBDs) each coordinating one zinc ion via cysteines arranged in a motif of the form CXXC-X_29_-CXXC [46]. Recombinant full-length E6 tends to precipitate upon tag-removal [47] and the aggregation propensity mainly resides in the amino-terminal zinc-binding domain of E6 [48]. Notably, continued efforts have recently born out fruits and resulted in NMR spectroscopy derived solution structures of the N-terminal and of the C-terminal zinc-binding domain of HPV 16 E6 [49], [50]. However, all of these E6 constructs contained mutations in order to ensure sample monodispersity and to prevent aggregation [50]. Unfortunately, these mutations abolish the hallmark property of full-length E6 to bind and degrade p53 [49], [51]. In particular, the F47R mutation prevents dimerization of E6 [50]. Dimerization (and possibly further aggregation), however, may be required for full E6 activity [50], [51]. The interaction of E6-derived short 6 or 7mer peptides, respectively, with PDZ domains of hDlg was previously elucidated by X-ray crystallography (PDZ2, PDZ3) [52] and NMR spectroscopy (PDZ2) [53].

Up to now, no structural data on a wild-type HPV E6 are available. So we reasoned that addressing wild-type E6 proteins of high-risk types other than HPV 16 could on the one hand lead to a structure of a wild–type E6 and on the other hand shed light on structural similarities and differences among the E6 proteins including their interaction with target proteins. Here we determined the solution structure of the wild-type C-terminal zinc-binding domain of E6 derived from the high-risk HPV 51 and unraveled the structural basis of its interaction with the PDZ domain 2 of hDlg.

## Results

Assessment of solubility of amino-terminally His_6_-tagged, recombinant E6 constructs derived from the high-risk types 16, 18, 26, 31, 45, 51, 59 and 97 as well as for the non-tumorigenic cutaneous type 1a was performed after bacterial expression in presence of 10 µM of Zn^2+^ as solubility of E6 constructs is dependent on low µM concentrations of zinc [54]. The results are summarized in Table S1. None of the wild-type sequence full-length E6 constructs nor their respective N-terminal zinc-binding domain (ZBD) turned out to be soluble (Table S1). However, all C-terminal ZBDs of high-risk E6 proteins were at least partially soluble except for HPV 59 which could not be expressed at all although codon-optimized DNA sequences for expression in *E. coli* had been employed (Table S1). The soluble constructs are denoted as E6Z2 (*e.g.* 51Z2 stands for the second, i.e. C-terminal, ZBD of HPV 51 E6).

**Figure 1:**
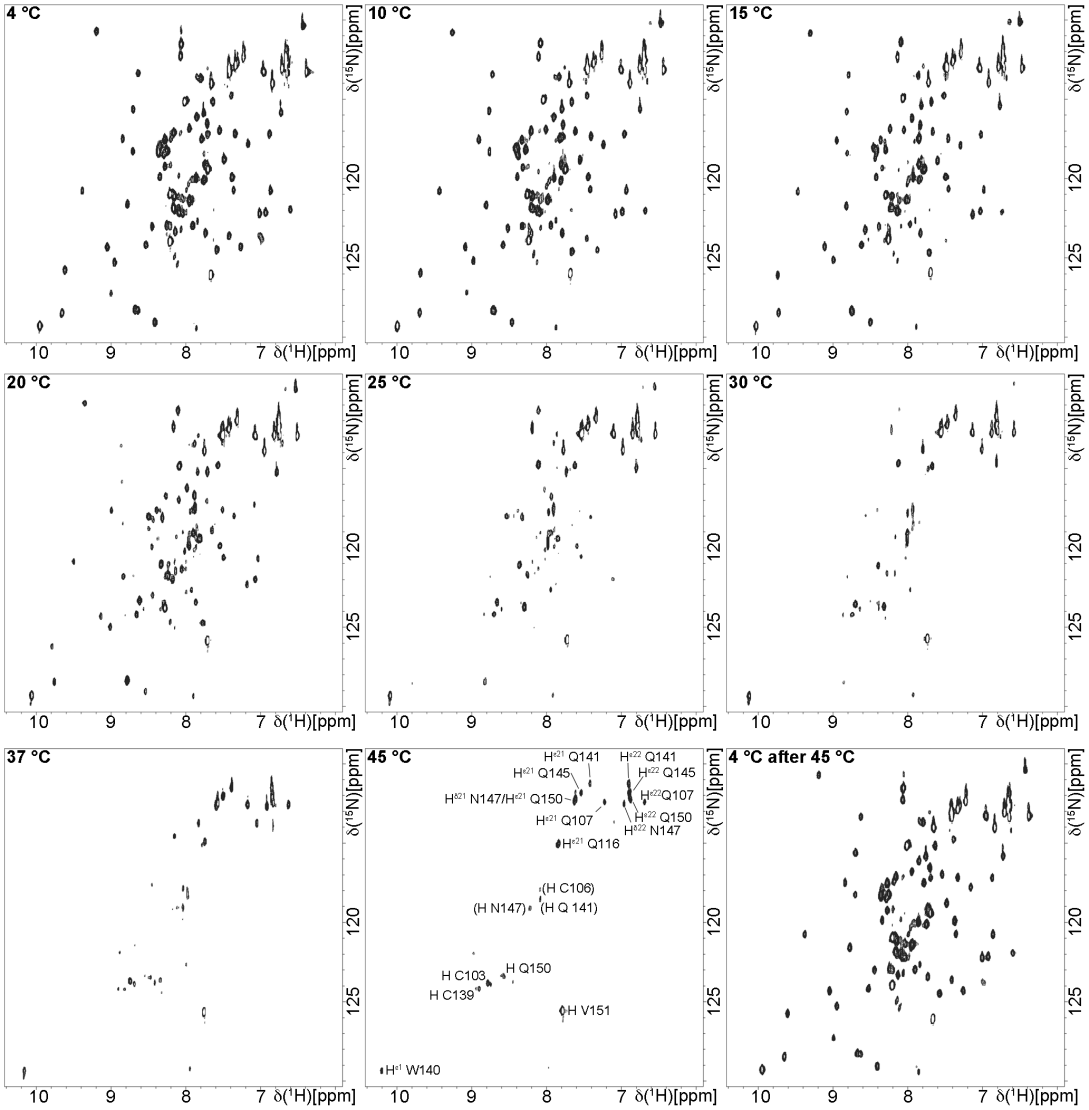
Temperature sensitivity of the HPV 51 C-terminal zinc-binding domain 51Z2. Nine [^1^H-^15^N]-HSQC spectra of an 51Z2 sample were recorded with identical spectral parameters and only the temperature was increased as indicated in the spectral plots. Residues that were still observable at 45°C are indicated in the 45°C spectrum. The assignment for the 45°C signals was based on the assignment for 10°C sample temperature (Figure 2) by tracking peak positions with increasing temperatures. Tentative assignments which were not unambiguous are given in parentheses. One control shows the 51Z2 spectrum after returning to the start temperature.

The soluble E6Z2s (Table S2) were purified and tested for monodispersity as detailed in the SI. For constructs that appeared to be monodisperse, [^1^H,^15^N]-HSQC spectra were recorded (see SI for details). If such a spectrum contained an acceptable signal distribution, homogeneous peak intensities and a signal number consistent with the expected number of observable amide groups, the respective protein was considered amenable to further NMR spectroscopic analysis. In the end, only 26Z2 and 51Z2 fulfilled these criteria (Table S2). Since 26Z2 exhibited signs of unfolding as indicated by spectral changes after a few days (Figure S1, Table S2), we finally concentrated our efforts on 51Z2. Results of the initial characterization of this E6 domain are presented in Figure S2. [^1^H,^15^N]- HSQC spectra of 51Z2 recorded at increasing temperatures from 4°C to 45°C resulted in reversible signal disappearance above 20°C (Figure 1). Only side chain signals, a few signals from backbone amides of the flexible C-terminal E6 tail and of the cysteines involved in zinc coordination were detected at elevated temperatures (Figure 1).

**Figure 2:**
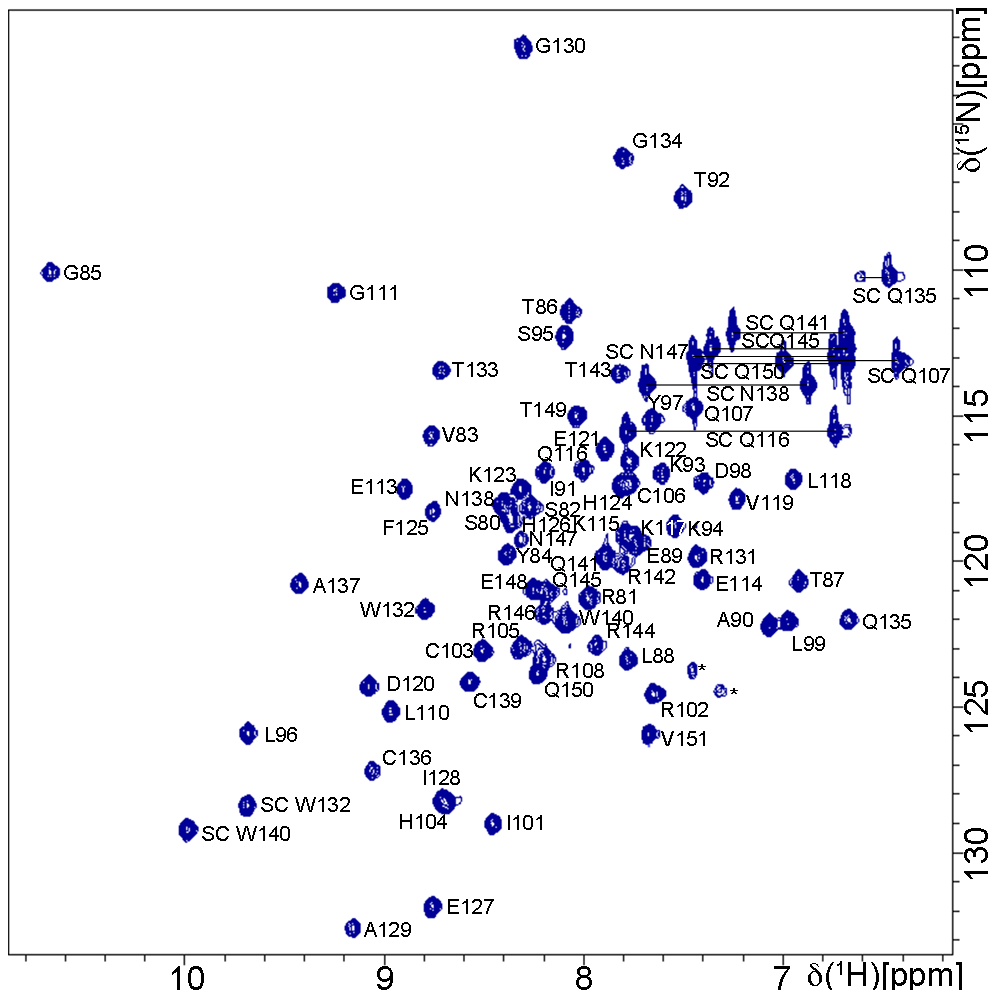
Resonance assignment of 51Z2. [^1^H,^15^N]-HSQC of 1.1 mM 51Z2 with indicated assignments and residue numbering according to full-length E6 sequence. Side chain signals are labeled with SC, and pairs of SC signals are linked by horizontal lines. Two starred resonances: folded signals probably representing arginine-side chains that could not unambiguously be assigned. Experimental details for this spectrum can be found in Table S3. The assignment has been deposited in the BMRB (Accession number: 18967).

Resonance assignment (Figure 2) and Nuclear Overhauser Effect (NOE) NMR-spectroscopy was carried out at 10°C (Table S3). The resonance assignment for customarily NMR observable nuclei is virtually complete for the E6 residues (see SI for details). Subsequently, 1501 NOEs, torsion angle constraints derived from 63 experimentally determined HNHA ^3^J-couplings as well as TALOS+ derived constraints (Table 1, SI) were utilized for distance geometry-based structure calculation with CYANA [55]. The calculated final structures were subjected to energy minimization in water employing CNS [56]. Resonance assignment and the structure of 51Z2 have been deposited in the BMRB and PDB databases (entries 18967 and 2M3L, respectively). The structural statistics are given in Table 1.

**Table 1:** Statistics for the 20 best 51Z2 and hDlgPDZ2-E6CT11 peptide complex structures.^a^ [Table-fn nt101]

Structure	51Z2	hDlgPDZ2-E6CT11
NOE-based distance restraints	1501	2760
intra-residual (|i−j| = 0)	306	425
sequential (|i−j| = 1)	333	693
medium-range (2≤|i−j|≤4)	338	479
long-range (|i−j|≥5)	524	1163^b^
inter-chain	not applicable	162
Zinc geometry constraints	2×9	not applicable
(upper and lower)		
Restrained H-bonds	15	32
Dihedral angle restraints		
Φ/Ψ	71/73	100/99
^ 3^J HNHA coupling derived	63	97
χ_1_	60	78
Leu χ_2_	0	6
Ile χ_21_	0	5
CYANA target function	0.26–1.39	0.81–1.58
R.m.s.d. after water refinement [Å]		
Total^c^	2.991	1.433
Ordered (backbone)^c^	0.623	0.496
Ordered (heavy atoms)^c^	1.255	0.964
Ramachandran plot [%]		
most-favored	85.3	82.6
additionally allowed	14.7	16.3
generously allowed	0	1.2
disallowed	0	0.0

a Methods detailed in the Supplementary Information.

b including the intermolecular (inter-chain) distance restraints.

c Residue numbers 80 to 151 refer to position of the full-length HPV 51 E6 protein (UniProtKB entry: P26554), while residues 318 to 406 refer to positions of the full length hDlg protein (UniProtKB entry: Q12959). Flexible non-native residues (amino-terminal GSHM of 51Z2 and carboxy-terminal His_6_-Tag of hDlgPDZ2) were not included in this structural statistics. For 51Z2 r.m.s.d. calculations include residues 80–151 or 80–140, respectively (see text). For the hDlgPDZ2-E6CT11 complex, r.m.s.d. calculations include residues 318–406 (hDlg) and 141–151 (E6) or 318–406 (hDlg) and 143–151 (E6), respectively.

**Figure 3:**
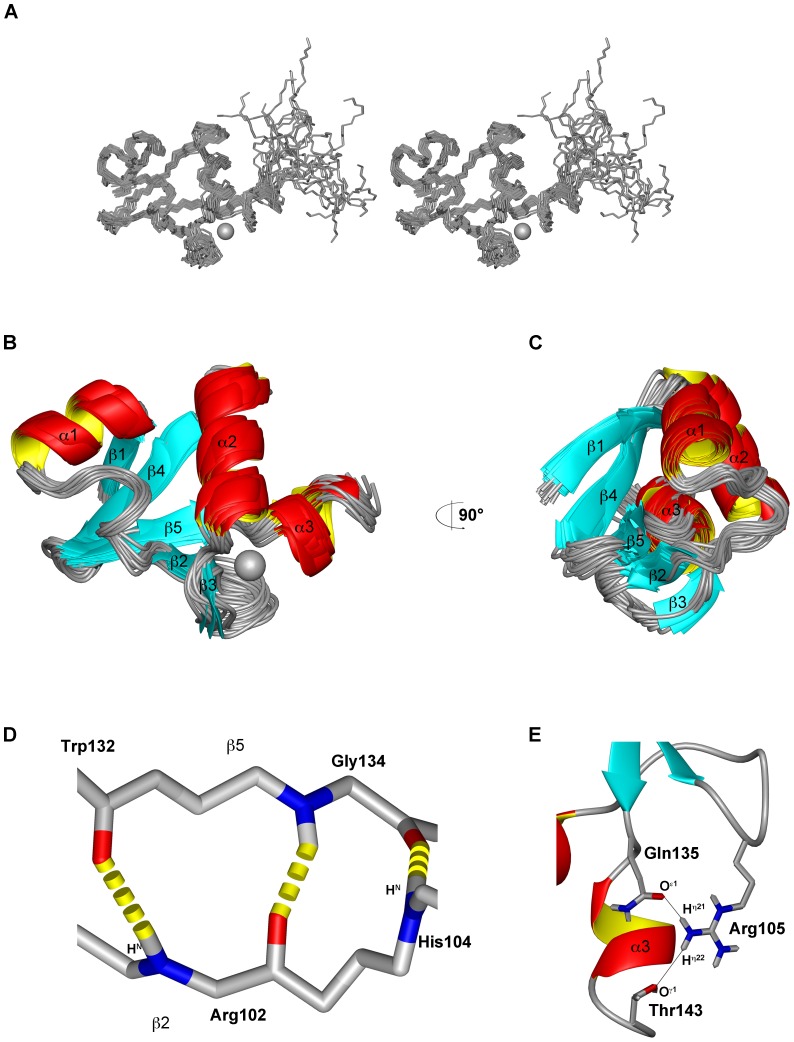
Solution structure of the C-terminal zinc-binding domain of HPV 51 E6 (51Z2). The bound zinc ion is represented as grey sphere. **A** Stereo view of the 51Z2 bundle of the 20 structures with the lowest energy after CNS refinement. **B** Ribbon view of the structural ensemble as in **A** with labeled secondary structure elements. The less ordered C-terminal residues 141–151 are omitted for clarity. **C** represents the rotated ensemble of **B**. **D** Backbone hydrogen bonds between residues on β2 and β5 strands stabilize the arrangement of both 51Z2 β-sheets. Side chains omitted for clarity. **E** The bidentate H-bond involving Arg105, Gln135 and Thr143 stabilizes the C-terminal α3 helix. The coordinates of 51Z2 have been deposited (PDB: 2M3L).

The calculated structural ensemble (Figure 3a) exhibits a backbone r.m.s.d. of 0.62 Å for the structured 51Z2 region (residues 80 to 140 of the full length E6) while the backbone r.m.s.d. drops to 2.37 Å when including the less ordered E6 C-terminus (residues 141–151, numbering according to full-length sequence). This C-terminus gave rise to only few NOEs consistent with a less ordered organization. Backbone torsion angles are located exclusively in most favored (85.3%) and additionally allowed (14.7%) regions of the Ramachandran plot (Table 1). The methyl groups of Ile88 show significantly upfield-shifted resonances (H^δ1^: −0.628 ppm; H^δ2^: −1.296 ppm). In the calculated structure, Ile88 H^δ2^ is located directly above the aromatic ring of Trp132, while Ile88 H^δ1^ is proximal to the aromatic ring of Phe125. Thus, ring current effects [57] explain the observed upfield-shift of these resonances and are consistent with the presented 51Z2 structure.

51Z2 exhibits a β1α1β2β3α2β4β5α3 topology (Figure 3a–c). All α-helices are located on one side of 51Z2, while all β-sheets are juxtaposed on the opposite hemisphere of the domain (Figures 3b and 3c). The closest-to-mean structure of the well-folded 51Z2 portion is presented in Figures 3b and 3c. The N-terminal part of α3 carries regular helical geometry, while its C-terminal residues arrange as 3_10_ helix. One anti-parallel β-sheet on 51Z2 is composed of the strands β1, β4 and β5, stabilized by charge-charge interactions and side chain hydrogen bonds, *e.g.* from R81 H^η12^ to E127 O^ε1^. A second short anti-parallel β-sheet consisting of β2 and β3 is arranged almost perpendicular to the first sheet. This allows for the formation of hydrogen bonds between R102 H^N^ and W132 O, H104 H^N^ to G134 O and G134 H^N^ to R102 O which stabilize the arrangement of both β-strands (Figure 3d). A turn-like structural element, located between α1 and β2, is stabilized by ionic interactions and the correlated formation of a hydrogen bond between the side chains of residues K94 H^ζ2^ and D98 O^δ2^ and additionally fixed by a hydrogen bond between L96 H^N^ to E89 O^ε1^. A bi-dentate hydrogen bond between Q135 O^ε1^ and R105 H^η21^ as well as R105 H^η22^ to T143 O^γ1^ stabilizes the spatial arrangement of the turn of the second β-sheet and the third α-helix (Figure 3e).

**Figure 4:**
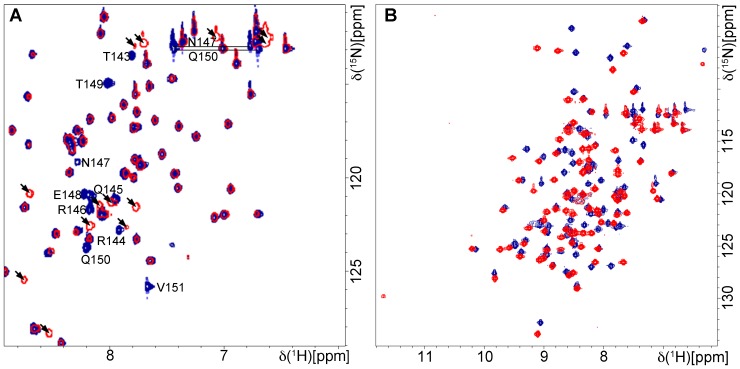
Interaction of 51Z2 with hDlgPDZ2. 100 µM of ^15^N labeled sample without (blue) or with (red) 3 fold excess of unlabeled interaction partner. **A** Labeled 51Z2 and unlabeled hDlgPDZ2. Only the spectral region with perturbed resonances is shown for clarity. The signal intensity for the nine C-terminal 51Z2 residues as well as resonances of side chains N147 and Q150 is diminished and a corresponding number of new signals is observed in presence of hDlgPDZ2 (arrowheads). The assignments (BMRB entry 18967) of perturbed residues on free 51Z2 are indicated. **B** labeled hDlgPDZ2 and unlabeled E6CT11, derived from the flexible 51Z2 C-terminus including the E6 residues perturbed upon binding. Resonance assignments of perturbed residues of hDlgPDZ2 are not indicated for clarity, since virtually all resonances show chemical shift differences. For resonance assignment of the hDlgPDZ2 complexed with the E6CT11, see ([60]; BMRB entry 17942).

**Figure 5:**
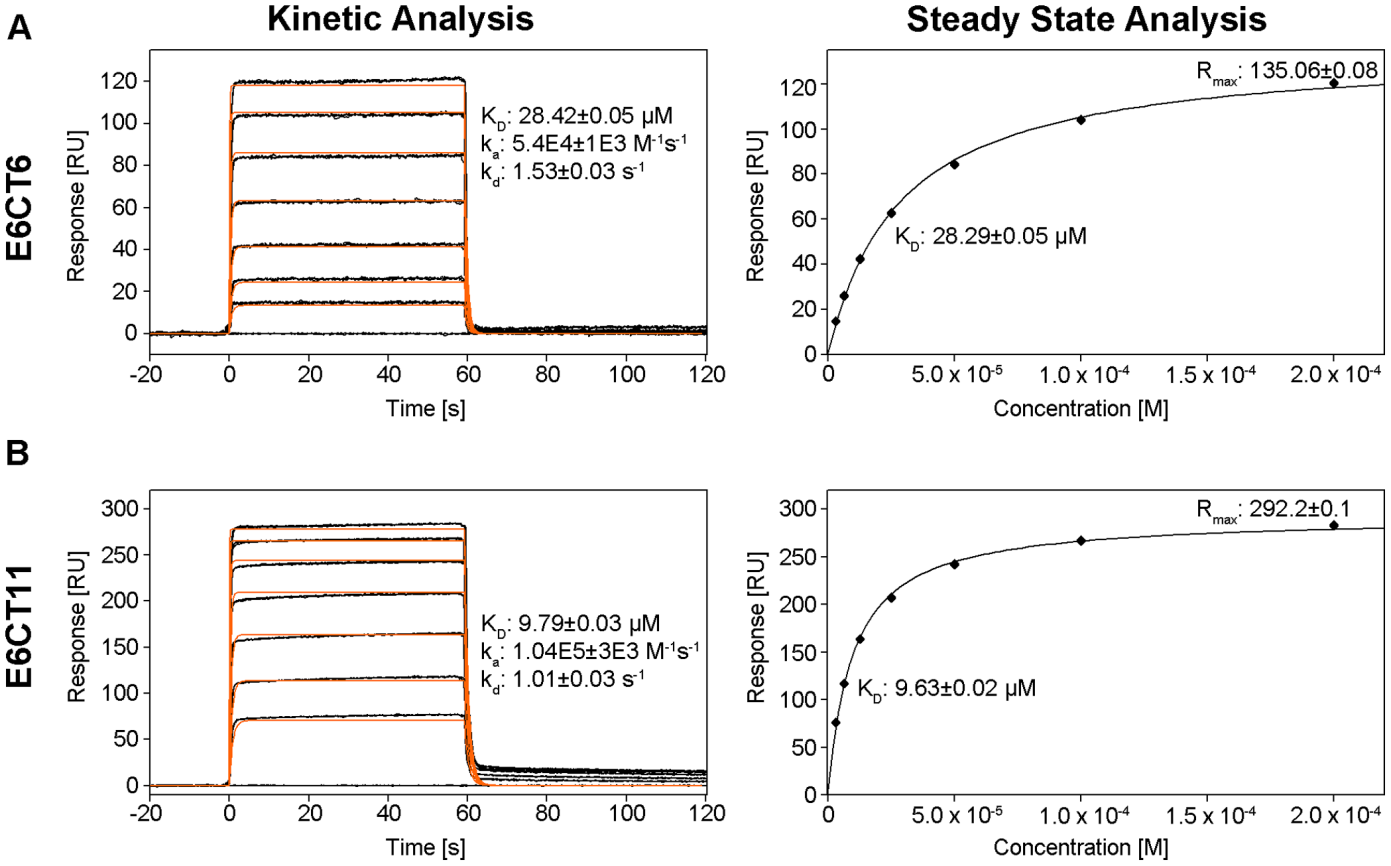
51Z2 derived E6CT6 peptide versus E6CT11 peptide binding to hDlgPDZ2. SPR data. **A** E6CT6, **B** E6CT11. Sensorgrams of E6CT6 and E6CT11 binding injected in triplicate (black lines) are shown overlaid with the best fit derived from a 1∶1 interaction model including a mass transport term (orange lines). Peptide concentrations of 3.125, 6.25, 12.5, 25, 50, 100 and 200 µM are shown. The binding parameters were obtained by kinetic analysis of association and dissociation phases (left panels) or by steady state analysis (right panels) utilizing signals of plateaus depicted in the corresponding left panel.

51Z2 contains a carboxy-terminal PDZ-BM and the full-length protein causes the degradation of hDlg *in vitro* [32]. Since HPV 16 and 18 E6 interact with hDlgPDZ2 and because the available structural hDlg-E6 interaction data contained only short E6 peptides of 6 or 7 residues [52], [53], [58] we investigated how 51Z2, representing a complete and wild-type domain of a high-risk HPV E6, interacts with hDlgPDZ2. An interaction is observed, as several chemical shifts of amide groups arising from the C-terminus of 51Z2 were perturbed in presence of hDlgPDZ2 (Figure 4a). Surprisingly, the perturbation affected the C-terminal nine E6 residues (143 to 151), more than anticipated from either the canonical PDZ-BM or from the already published hDlgPDZ2-E6 peptide complex structures [52], [53]. From the gradual disappearance of resonances of the unbound 51Z2 and reappearance of the same number of resonances in the bound state of this domain we concluded that this interaction occurs within the slow exchange regime on the NMR time scale. Importantly, no further E6 resonances experienced chemical shift perturbation, clearly indicating that the interaction with the hDlgPDZ2 is confined to the disordered C-terminal region harboring the PDZ-BM of HPV51 E6. When titrated with an 11mer peptide representing the complete E6 disordered C-terminus (Ac-QRTRQRNETQV, corresponding to HPV 51 E6 residues 141 to 151, further referred to as E6CT11), the hDlgPDZ2 also showed significant spectral changes (Figure 4B). Moreover, hDlgPDZ2 bound to E6CT11 exhibited additional chemical shift perturbations when compared to hDlgPDZ2 in complex with an N-terminally truncated 6mer peptide (Ac-RNETQV, further referred to as E6CT6). In order to assess the affinity of the two different representative peptides of the E6 C-terminus, surface plasmon resonance (SPR) with hDlgPDZ2 and the E6CT11 and E6CT6 peptides, respectively, was performed. The SPR data indicate a contribution to binding of the additional residues as the E6CT11 binds with higher affinity to hDlgPDZ2 than the E6CT6 (K_d_ 9.6 µM *versus* 28.3 µM, respectively; Figure 5). Owing to fast association and dissociation (Figure 5 left panels), a kinetic analysis of the SPR data is more error-prone. The kinetically derived K_d_ values, however, are virtually identical to the values of the steady state analyses (Figure 5 right panels). The kinetic analysis reveals that the association is approximately 2-fold faster and dissociation 1/3^rd^ slower for the E6CT11 as compared to the E6CT6, resulting in a total difference in affinity by a factor of 3.

**Figure 6:**
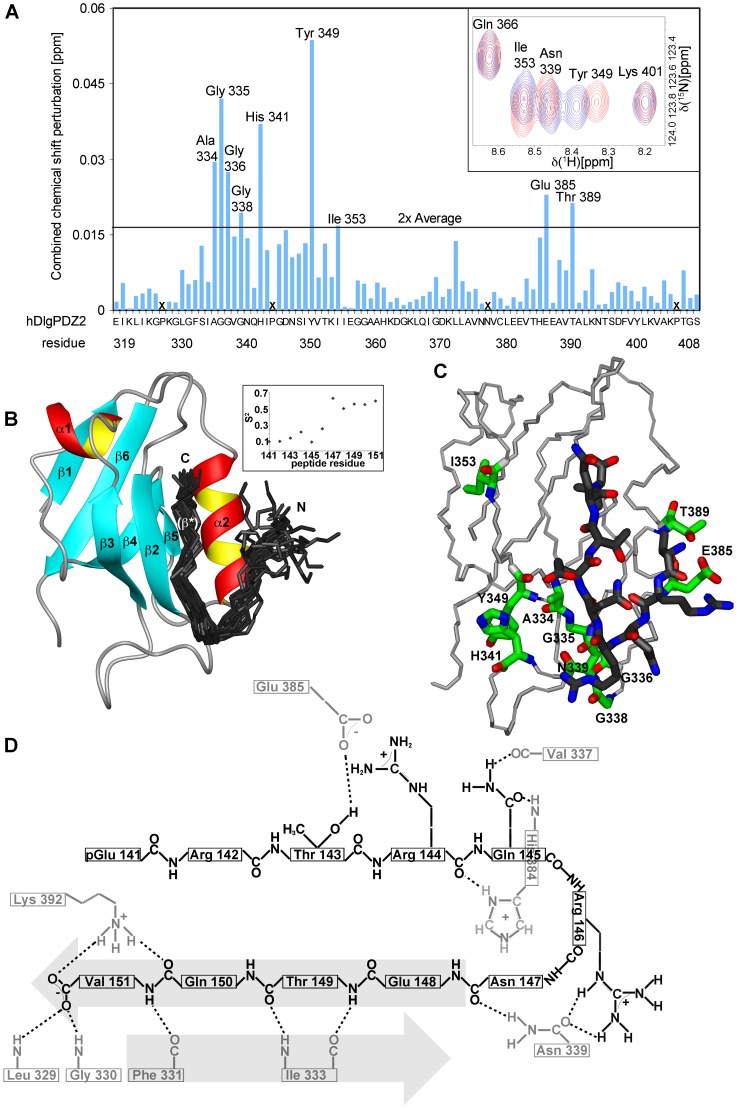
Interaction of E6CT11 with hDlgPDZ2. **A** Combined ^1^H and ^15^N chemical shift perturbation (as detailed in SI) of 100 µM hDlgPDZ2 in complex with 300 µM E6CT11 peptide versus 300 µM E6CT6 peptide. Residues without observable amide shifts are denoted with X. The inset of a region of the corresponding HSQC spectra show unperturbed as well as perturbed signals. Red contours: hDlgPDZ2 complexed with E6CT11, blue contours: hDlgPDZ2 complexed with E6CT6. Note that the side chain amide signals of Asn339 were also perturbed by more than 2× the average value. **B** Structure of the hDlgPDZ2-E6CT11 complex. The bundle of 20 best E6CT11 structures (residues 141 to 151, dark grey) is shown together with a ribbon of the closest-to-mean hDlgPDZ2 structure (hDlg residues 318–406). Peptide structures were fitted to residues 143 to 151 and the termini are indicated. Secondary structure elements are labeled. The boxed inset depicts per-residue backbone order parameters of the complexed E6CT11 peptide. **C** Details of the hDlgPDZ2-E6CT11 complex. hDlgPDZ2 backbone trace depicted in light gray. PDZ side chains (heavy atoms) of residues showing most perturbed combined amide group chemical shifts (backbone and Asn339 side chain; Figure 6a) are depicted in green and labeled, while the closest-to-mean E6CT11 peptide structure (heavy atoms, residues 143–151) is presented in dark gray. **D** Schematic depiction of intermolecular hydrogen bonds and salt bridges in the clostest-to-mean complex structure. Indicated side-chains start at the Cβ atom. Hydrogen bonds are indicated as dashed lines. Secondary structure elements β* and β2 are emphasized by arrows; hDlgPDZ2 residues appear gray, while peptide residues are depicted in black.

In order to elucidate this difference in structural terms, the hDlgPDZ2-E6CT11 complex structure was determined exploiting stable isotope labeled E6CT11 (experimental details see Table S4). During the production of ^13^C and ^15^N labeled E6CT11 using the intein system, spontaneous cyclization of its amino-terminal glutamine to pyroglutamate was observed [59]. This covalent automodification, however, has no bearing on complex formation as shown earlier [60]. Completion of the resonance assignment and structure determination of hDlgPDZ2 complexed with the E6CT11 (for details see SI, BMRB entry 17942, [60] and PDB ID: 2M3M) allowed for the identification of hDlg residues additionally affected by the extended peptide (Figure 6a). In the final complex structure (Figures 6b and 6c), the most perturbed residues (backbone amide groups of A334, G 335, G336, H341, Y349, E385, T389 and side chain amide group of N339) are situated in a region of the PDZ domain that is close to the peptide binding region (Figure 6c). Only one additionally perturbed backbone amide of I353 is located far away from the E6CT11 binding region of hDlgPDZ2. Of particular note are the perturbed residues E385 and T389 of hDlgPDZ2, which are contacted by the amino-terminal peptide residues T143 and R144 that were lacking in the previously characterized hDlgPDZ2-E6 complexes [52], [53].

The solution structure of the E6CT11 complexed hDlgPDZ2 (Table 1; Figures 6b and 6c) forms a compact domain with a β1β2β3α1β4β5α2β6 topology, in line with the topology of hDlgPDZ2 complexed with shorter peptides [52], [53] and consistent with the common fold of PDZ domains [61]. The core of hDlgPDZ2 is formed by the side chains of a number of hydrophobic amino acids of the β-strands (I320, L322, L329, F331, I333, V350, L365, L371, L400, V402) and L391 emanating from the central α-helix. In complex with the E6CT11 peptide, this α-helix can be regarded as an anchoring element by presenting its charged amino acids E385 and K392, located at the α2 N- and C-terminus, respectively, into the solvent. Of the two peptide residues attaching to this anchor, residue Q150 is located in the C-terminal part of the peptide (E148-V151) essentially forming an additional β-strand (β*) anti-parallel to strand β2 of hDlgPDZ2 upon complexation. The second residue of the peptide, R142, could contribute to short-lived side chain charge-charge interactions with E385 of hDlgPDZ2. However, the R142 of E6 is located in the very N-terminus of the peptide. As deduced from the order parameters (Figure 6, inset), this entity experiences a higher motional freedom than the C-terminal part of the E6CT11 peptide which is ‘immobilized’ as an integral part of the hDlgPDZ2 β-sheet architecture. For residues T143 and R144 of E6, however, well-defined side chain interactions to T389 and E385 of hDlgPDZ2 are present, consistent with the observed chemical shift perturbation for these residues (Figures 6a and 6c). The R146 side chain also contacts the PDZ domain at residues G338 and N339, the amide resonances of which are perturbed as well in the complex.

In conclusion, the main contribution of binding of the E6CT11 peptide to the hDlgPDZ2 is the formation of a stable and rigid additional β-strand. Overall, the peptide forms a kinked shape-complementary structure allowing its C-terminus to align anti-parallel to the β-strand 2 of the PDZ domain while the very N-terminus of the peptide is separated by a turn-like structure and thus a clash with the turn of the hDlgPDZ2 involving residues V337-N339 is prevented. Additional contacts of residues that are located N-terminal to the canonical PDZ-BM which is located on the β* strand establish further contacts between E6 and the hDlgPDZ2, involving charge complementarity: Arg 144 on E6 interacts with E385 on hDlgPDZ2 (Figure 6c). These additional contacts are consistent with the increased affinity as seen in the SPR experiments (Figure 5).

## Discussion

We set out to characterize a full length, wildtype E6 structure. The result of these efforts is the structure of the C-terminal zinc-binding domain (ZBD) of HPV 51 E6 (residues 80 to 151; 51Z2) and represents the first structural characterization of a non-mutated HPV E6 domain. The International Agency for Research on Cancer classifies HPV 51 as high-risk [62] and in HPV 51 infected cells, p53 and hDlg are degraded [32], [63]. All wild-type, high-risk E6 constructs containing the amino-terminal ZBD tested here were prone to aggregation and insolubility. Dimerization and aggregation propensity of HPV 16 E6 resides mainly in the amino-terminal ZBD [48], [50]. In combination with our solubility screening this strongly suggests that dimerization and/or aggregation mediated by the E6 amino-terminal domain may be a property shared among high-risk E6 proteins.

**Figure 7:**
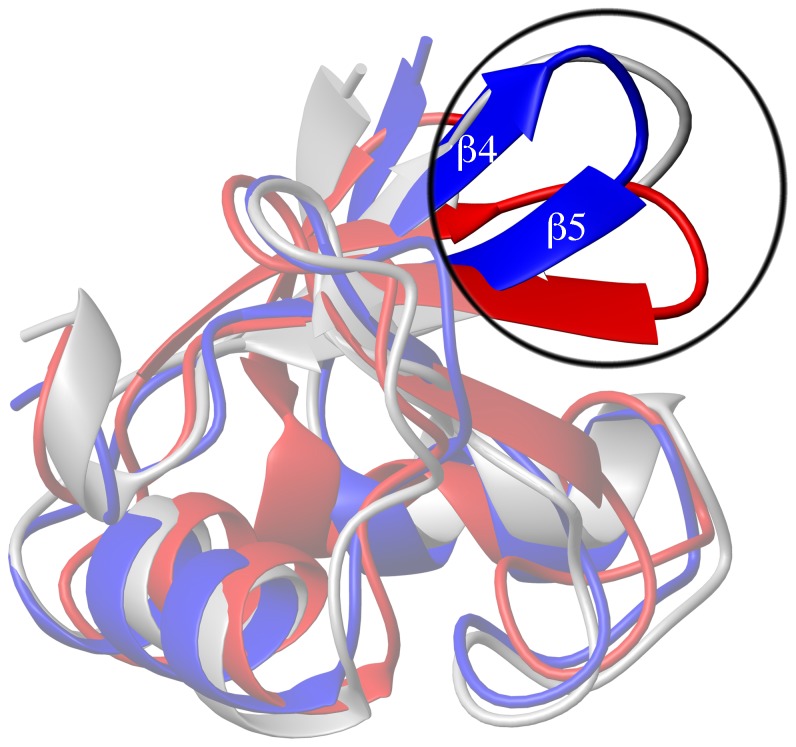
Superimposition of 51Z2 to other E6 structures. Superimposition (details: see SI) of the 51Z2 closest-to mean structure (folded part, residues 80–140, blue) onto the corresponding regions of HPV 16 E6 (red, PDB ID 2LJZ, r.m.s.d. 2.27 Å) and BPV E6 in complex with the LD1 motif of paxillin (gray, PDB ID 3PY7, paxillin omitted for clarity, r.m.s.d. 1.61 Å). The overall topology is conserved. Notably, the β4 and β5 strands and their connecting loop of 51Z2 and BPV position similar, while for HPV 16 E6, the corresponding region orients differently (upper right corner; encircled and highlighted).

The hydrodynamic radius and secondary structure content estimated from circular dichroism spectroscopy of the wild-type 51Z2 are consistent with the final, monomeric 51Z2 structure. The reversibly disappearing 51Z2 backbone signals above 20°C *in vitro* suggest protein motion at a timescale that causes line broadening at higher temperatures, whereas the zinc coordinating residues remain rigid. These findings derived from a wild-type E6 domain allow us to suggest that in cervical epithelial cells, where the E6 protein is expressed at 37°C, this particular domain and potentially the entire E6 protein may be more malleable *in vivo* than their published structures suggest at first glance. As the fold is re-adopted upon cooling down (Figure 1), it could be argued that the present low temperature conformation represents a low energy state and that this might be the conformation the C-terminal E6 domain adopts upon complex formation. The close structural match of 51Z2 to the corresponding domain of ligand-bound BPV E6 (Figure 7) supports this idea. The presence of E6 interactors such as hScrib and hDlg [64] or E6AP [65] stabilizes E6 *in vivo*, supporting our hypothesis that E6 interactors may stabilize an energetically favored E6 fold that might be less prone to proteolytic turnover *in vivo*. Consistent with this HPV 16 E6 interacts with p53 and experiences an increased stability *in vivo* in presence of commonly E6 binding LXXLL-containing peptides [66]. The malleability of E6 is reminiscent of intrinsically disordered proteins that undergo a disorder-to-order transition upon productive complex formation with specific ligands [67]. In the case of E6 this might be functional in the context of binding to the multitude of cellular E6 interaction partners [18] and further studies are needed to address the dynamic aspects of E6 plasticity (ZBD2; this paper) and dimerization (ZBD1; [50], [51]) of wild-type E6.

A structural comparison of the unbound, wild-type 51Z2 to the corresponding unbound, four-fold mutated ZBD2 of HPV 16 E6 and to the evolutionarily distant bovine papilloma virus 1 (BPV) E6 in complex with the LD1 motif of paxillin reveals an identical general topology for E6 (Figure 7). Thus, it is reasonable to assume a similar fold for the corresponding domains of at least other high-risk or even of all E6 proteins. To analyze this similarity in more detail, sequences of E6 shared by the HPV types for which there is reasonable evidence for their carcinogenicity [62] were aligned and conserved residues were identified (highlighted in Figure S3). In the following, residues are numbered according to their corresponding position in HPV 51 E6. Among the conserved residues, cysteines 103, 106, 136 and 139 coordinate the Zn^2+^ ion. Residues V83, L88, L96, L99, I101, L110, W132 and G134 form the E6 core. G85 constitutes the beginning of the first α-helix of ZBD2. Residues S82, Y84, T87, R102, P109, P112, E114, K115, R124, H126, I128, T149 and V 151 are solvent exposed and prone to contribute to binding of cellular targets of E6. We also note a hitherto unrecognized E6 sequence element involving the conserved residues P109 and P112 located on the E6 surface. This PXXP motif is present in all oncogenic E6 types (Figure S3) but rarely found in nononcogenic types (representative types see Figure S4; PXXP is present in HPV 7, 32, 40, 43, 91 E6). PXXP could be recognized by protein domains targeting proline-rich sequences (such as SH3; [68], [69]). The conserved, charged E6 residues E114 and K115 in spatial proximity to PXXP could further enhance binding affinity and specificity of this PXXP motif as observed for other SH3 interactions [68].

Interestingly, a direct correlation between E6 phylogeny and their protein-protein interaction networks exists [70], *i.e.* the target spectrum of closely related E6 is more similar than that of evolutionary more distantly related E6. Thus, although the E6 proteins may share a generally identical structural topology, subtle structural variations could explain the altered target spectrum of different E6 proteins [70], [71], strongly arguing in favor of structural characterization of further E6 proteins embedded in different interaction networks.

**Figure 8:**
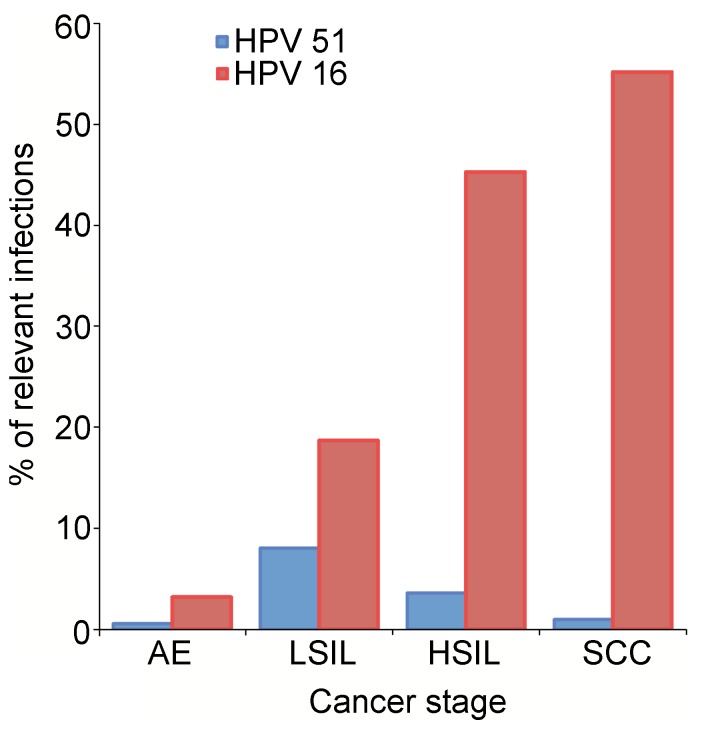
Comparison of HPV 51 and HPV 16 prevalence. Meta-analysis of prevalence of HPV 51 (blue) and HPV 16 (red) in asymptomatic epithelia (AE; [75]) and in (pre-)cancerous stages low-grade squamous intraepithelial lesions (LSIL; [76]), high-grade squamous intraepithelial lesions (HSIL; [4]) and squamous cell carcinoma (SCC; [4]). While the fraction of HPV 16 increases with severity of neoplasia, the HPV 51 fraction decreases after the LSIL stage.

Our analysis allows the identification of significant local structural differences between individual E6 proteins. Particularly, β4 and β5 of 51Z2 position differently as compared to the unbound ZBD2 of HPV 16 E6, but similar to the corresponding region of the only available full-length E6 structure in a complex, the BPV E6 bound to LD1 of paxillin (Figure 7). The conserved I128 (Figure S3) is a key residue for the E6-E6AP interaction [71], [72], [73] and a single I128T E6 mutation in the genome of the high-risk HPV 31 causes viral episome loss after a few cell passages [74]. In the unbound 51Z2 I128 resides on β4 that orients differently with respect to the HPV 16 E6 structure (*vide supra*). In 51Z2 I128 is proximal to the conserved residues H126 and S82 on β4 and β1, respectively. HPV 51 is, as HPV 16, frequently detected in premalignant neoplasias, but proportionally significantly less present in cervical cancers [4], [75], [76](Figure 8). In a three-stage model of carcinogenesis (initiation, promotion, progression), the later stages of cervical cancer progression are governed by E6 functions [77]. Therefore, the different E6 conformation in the region around I128, subtle as it is, could entail altered interaction properties and/or a change in relative positioning between E6 and LXXLL-containing proteins such as *e.g.* E6AP or paxillin. Since the E3 ubiquitin ligase E6AP is often involved in E6-mediated target degradation [22], [23], [78], an alteration of this particular interaction might have implications for the proteasomal degradation of other E6 interactors as well and may influence the fate of cells infected by different HPV types. This invites the speculation that the subtle difference in the structures of HPV 16 and 51 E6 contribute to the different prevalence in high-grade cervical lesions of these two HPV types. Of course, other regions or properties of E6 or even differences between the respective E7 proteins are likely to be also relevant to the difference in oncogenicity of the two high-risk HPV types and to clarify this will require substantial but potentially fruitful future work. During revision of this manuscript, the publication for the complexed BPV E6 structure (PDB 3PY7; [79]) became available. The paper also describes the structure of a mutated HPV 16 E6 in complex with an E6AP-derived peptide. There, the complexed ZBD2 of HPV 16 E6 adopts a conformation similar to the conformation of the unbound 51Z2 but different to the conformation of the unbound HPV 16 ZBD2. Our structure interpretation as elaborated above is consistent with these findings.

51Z2 in the unbound state adopts a globular fold with a solvent exposed and flexible C-terminus (Figures 3 and S3) comprising the PDZ-BM of high-risk HPV, including the conserved T149 and V151 PDZ-BM key residues [31]. This structure provides for an E6 C-terminus accessible for binding to target PDZ domains and the HPV 51 E6 PDZ-BM binds to hDlgPDZ2 (Figures 4, 5 and 6). The structure of the E6CT11-bound hDlgPDZ2 domain is similar to the available structures of hDlgPDZ2 in complex with shorter peptides [52], [53]. However, as a single mutation on a PDZ-BM dramatically alters viral virulence in a different context [37], a full analysis of all E6 residues involved in this PDZ binding appeared indicated. The previously available structures of hDlg PDZ domains complexed with E6-derived peptides contained the 4 most C-terminal residues forming the canonical E6 PDZ-BM and up to three additional residues [52], [53]. Our analysis of the 51Z2 interaction with hDlgPDZ2 by way of SPR and subsequent structure determination reveals that the C-terminal 9 E6 residues contribute to the interaction. Importantly, presence of residues N-terminal to the canonical PDZ-BM significantly increase PDZ-binding affinity (Figure 5). Using a library of synthetic peptides it was shown that optimal substrate specificity and affinity of several PDZ-domains requires 9 residue peptides [80]. This study included all three PDZ domains of murine Dlg that carry a greater than 98% sequence identity with the hDlg PDZ domains. Contribution of E6 residues upstream of the canonical PDZ-BM was also observed for the MAGI1 PDZ1 interaction [81]. Yet in that system, 2 of 8 MAGI1 PDZ1 contacting E6 peptide residues interact with a region outside the canonical PDZ-domain. In our system, however, at least nine E6CT11 residues contact canonical PDZ-domain residues of hDlgPDZ2. Hence, ‘supernumeral’ residues outside the canonical E6 PDZ-BM establish specific contacts with PDZ domains in different ways be it *via* contacts to *bona fide* PDZ domain residues [this study] or to residues outside the classical PDZ domain [81]. Evidently, such interactions may fine-tune E6-PDZ interactions. Here, the E6CT11 and E6CT6 peptides bind with affinities of slightly below 10 µM and slightly above 28 µM to hDlgPDZ2. An affinity to PDZ-domains below 10 µM is exceptional; more often the affinity lies in the 10 to 100 µM range [82]. Thus, the charge complementarity (and additional contacts) of the E6CT11 guarantees for high affinity, which could allow E6 to successfully compete with cellular molecules for hDlg binding. As basic residues upstream to the canonical PDZ-BM are present on all high-risk E6 proteins (though they are not conserved position-specifically; Figure S3), the charge complementarity could be a general property of high-risk E6 proteins for binding to certain PDZ domains. Moreover, since viruses often target PDZ-domains [35], [36], the ‘affinity boost’ by charge complementarity could also be one mechanism of other viral PDZ-targeting proteins to be ‘better binders’ than their competing cellular proteins.

A crystal structure of the C-terminal 11 residues of APC (APC_CT11) in complex with hDlgPDZ2 reveals only one peptide per asymmetric unit containing in turn five PDZ domains. Here, only the C-terminal six APC peptide residues gave rise to interpretable electron density [83]. The non-visible peptide residues, however, apparently contributed to affinity in that complex. The hDlgPDZ2-E6CT11 structure might explain the findings of the hDlgPDZ2-APC_CT11 complex of [83]: the increased flexibility observed for the N-terminus of E6CT11 in the hDlgPDZ2 complex might also apply to the APC_CT11-hDlgPDZ2 complex, which in turn may have compromised a complete X-ray structural analysis of the APC peptide residues. In conclusion, here we provide a virtually complete structural rational for extended PDZ-BM PDZ interactions for the first time. Based on this, it is tempting to speculate that the mechanism of PDZ-domain binding with residues upstream of the canonical PDZ-BM may be a common strategy of the PDZ-BM harboring high-risk E6 proteins to increase substrate affinity and specificity. Moreover, interaction with an extended PDZ-BM may be a common mechanism of at least a subset of PDZ-domains [61]. For example, PDZ domains apparently employ long-range networks for fine-tuning their binding selectivity [84] and two mutations, one of which is located far away from the peptide interface, in a PDZ domain confer altered binding specificities to this domain [85]. The single hDlgPDZ2 residue I353 affected differentially after binding of E6CT11 *versus* E6CT6 and located far away from the peptide interface might have been perturbed due to the presence of such a long range network. This raises the question if the HPV 51 E6 extended PDZ-BM may even induce changes in places remote from the PDZ 2 of hDlg as the PDZ domains 1 and 2 of the hDlg-related PSD-95 are organized as a supramodule and retain their relative orientation in the context of the full-length protein [86]. Since PDZ1 and 2 of hDlg are, as the domains of PSD-95, connected by a short linker, such a supramodular organization can also be envisioned for hDlg PDZ1 and 2 as there is evidence that this PDZ1-2 region forms a single conformational and functional unit [87]. The additional E6 residues that contribute to PDZ2 binding do not reach into the hypothetical hDlg PDZ1-PDZ2 interface and would thus not interfer directly with formation of a hDlg PDZ1-2 supramodule as deduced from applying the PSD-95 PDZ1-2 structural model [86] to hDlg here (not shown). Hence it should be interesting to further investigate the E6–hDlg interaction by using extended hDlg constructs to cover aspects of long range networks and of the PDZ supramodular organization.

In summary, the first structure of a wild-type E6 domain and of the complex between its complete PDZ-BM and the PDZ domain 2 of hDlg revealed a number of E6 properties common for high-risk HPV E6 but also important differences between individual E6. The fully characterized interaction of E6’s extended PDZ-BM to one PDZ domain suggests future routes towards the elucidation of PDZ-supramodule E6 interaction. The implications of the E6 structural variations and the impact of the probable malleability of E6 *in vivo* with regard to interactions, such as *e.g.* with E6AP, may be addressed based on our findings in order to solve the puzzle of how the E6 proteins exert their malignant potential *via* interaction with a multitude of cellular targets.

## Materials and Methods

A detailed description of methods is provided in Text S1. Briefly, recombinant proteins were generated in *E. coli* BL21 (DE3). Cells were disrupted and protein solubility in cell-lysates was assessed. Soluble proteins were FPLC-purified and initially characterized by CD spectroscopy, dynamic light scattering, gel filtration chromatography and NMR spectroscopy. The affinity of E6-derived peptides to hDlgPDZ2 was elucidated utilizing Surface Plasmon Resonance. hDlgPDZ2 was generated by bacterial expression and FPLC purification. The generation of the labeled E6CT11 peptide was performed as previously described [59]. The purified, monomeric 51Z2 and the purified, monomeric hDlgPDZ2 in complex with purified E6CT11 were subsequently structurally characterized by standard solution state NMR techniques.

## Supporting Information

Figure S1 **Spectral changes of 26Z2 over time.** Freshly prepared ^15^N-labeled 26Z2 (250 µM) was subjected to [^1^H,^15^N]-HSQC NMR spectroscopy (blue con-tours). After 10 days at 4°C, the spectra (red contours) showed significant differences. Low peak-dispersion suggests an increased proportion of unfolded protein. Both spectra were recorded with 16 scans at a Bruker Avance III 750 MHz NMR spectrometer. Sample conditions were 135 mM NaCl, 45 mM L-Arg, 45 mM L-Glu, 9 mM DTT, pH 7.4, 4°C.
(TIF)

Figure S2 **Biophysical characterization of 51Z2.** For experimental details, see supplementary text 1. **A** Analytical gel-filtration. The chromatogram of 51Z2 run on a TSK gel G3000SWxl column is presented in blue, the column calibration is shown in orange with molecular weights of reference proteins indicated. 51Z2 (calculated MW 8.9 kDa) eluted as a 9 to 10 kDa sized protein indicating a monomeric state. **B** Dynamic light scattering. 51Z2 exhibits a hydrodynamic radius of 1.58 nm, which corresponds to an approx. 10 kDa sized protein assuming a globular shape. **C** Circular dichroism spectrum of purified 51Z2. **D** The secondary structure content of 51Z2 was estimated from the CD spectrum **C** using CDNN [88].
(TIF)

Figure S3 **Sequence alignment of oncogenic E6 proteins.** ClustalW2 [89] was utilized for alignment of the E6 proteins from oncogenic/possibly oncogenic HPV types (according to IARC, [62]). The figure was prepared using jalview [90]. Residues with a jalview-implemented conservation score [91] of 9 or higher were colored in green. The oncogenic HPV types [62] phylogenetically belong to the genus *alpha-papillomaviridae* and to the species indicated on the left table-side.
(TIF)

Figure S4 **Alignment of the high-risk HPV 51 E6 to representative low-risk and cutaneous E6 proteins.** The residues corresponding to conserved positions among high-risk types (Figure S3) are bracketed. The bracket color is green, if any residue present among the high-risk E6 proteins at that position is encountered (Figure S3) or red, if the residue is never observed at the respective position of any high-risk E6 protein.
(TIF)

Table S1 **E6 domain architecture and solubility of recombinant E6 constructs.**
(PDF)

Table S2 **Final screening results of E6 constructs for NMR spectroscopy.**
(PDF)

Table S3 **Details of NMR experiments and samples for the 51Z2 structure determination.**
(PDF)

Table S4 **Details of NMR experiments and samples for the hDlgPDZ2-E6CT11 complex structure determination.**
(PDF)

Text S1 **Methods.**
(PDF)
