# Supplementary figures and images for: Structural Insights into a Wildtype Domain of the Oncoprotein E6 and Its Interaction with a PDZ Domain
Source: PLoS One. 2013 Apr 30;8(4):e62584. doi: 10.1371/journal.pone.0062584 (PMC3640046; doi:10.1371/journal.pone.0062584)

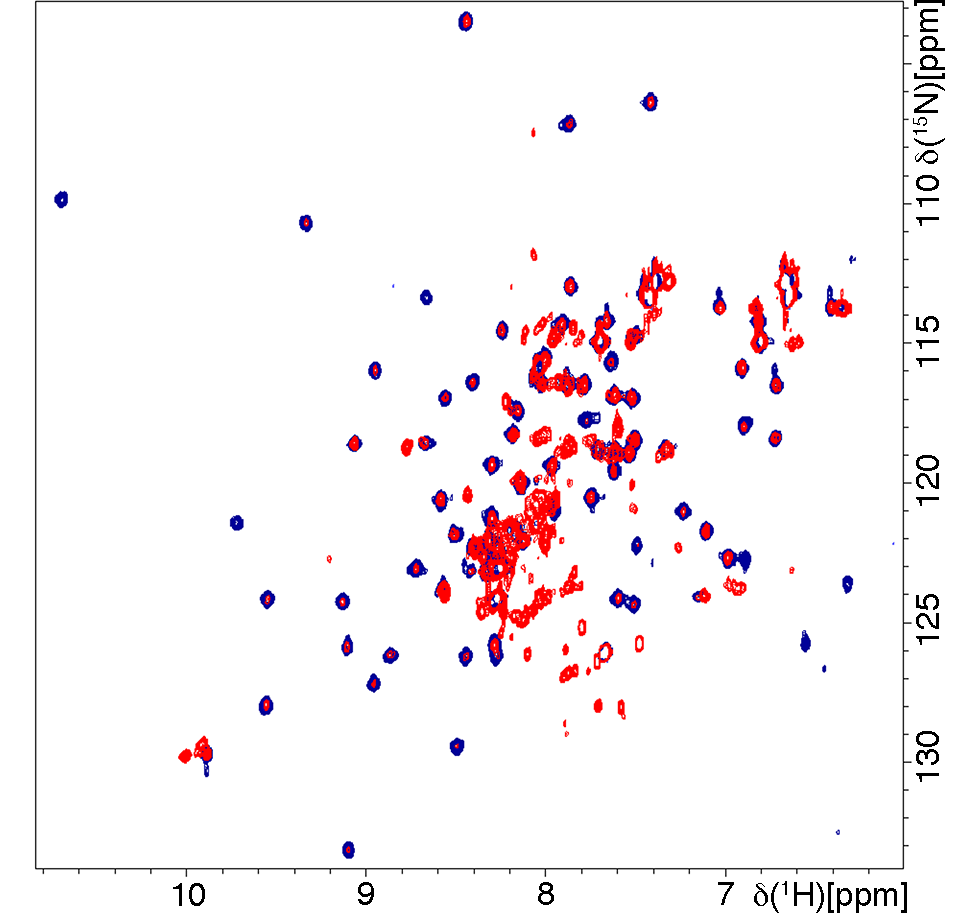

Supplement: Figure S1 — Spectral changes of 26Z2 over time. Freshly prepared 15N-labeled 26Z2 (250 µM) was subjected to [1H,15N]-HSQC NMR spectroscopy (blue con-tours). After 10 days at 4°C, the spectra (red contours) showed significant differences. Low peak-dispersion suggests an increased proportion of unfolded protein. Both spectra were recorded with 16 scans at a Bruker Avance III 750 MHz NMR spectrometer. Sample conditions were 135 mM NaCl, 45 mM L-Arg, 45 mM L-Glu, 9 mM DTT, pH 7.4, 4°C. (TIF) [file pone.0062584.s001.tif]

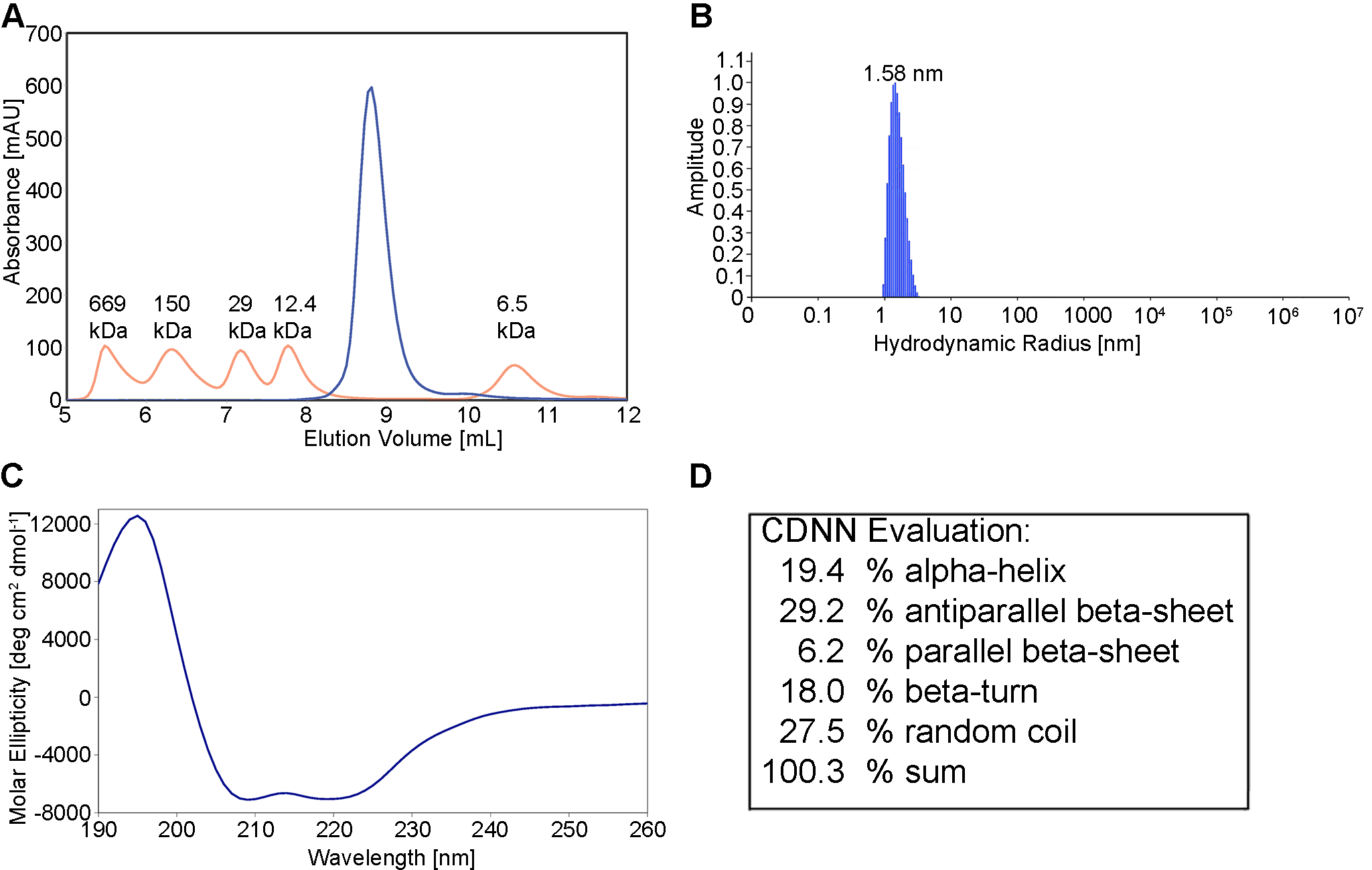

Supplement: Figure S2 — Biophysical characterization of 51Z2. For experimental details, see supplementary text 1. A Analytical gel-filtration. The chromatogram of 51Z2 run on a TSK gel G3000SWxl column is presented in blue, the column calibration is shown in orange with molecular weights of reference proteins indicated. 51Z2 (calculated MW 8.9 kDa) eluted as a 9 to 10 kDa sized protein indicating a monomeric state. B Dynamic light scattering. 51Z2 exhibits a hydrodynamic radius of 1.58 nm, which corresponds to an approx. 10 kDa sized protein assuming a globular shape. C Circular dichroism spectrum of purified 51Z2. D The secondary structure content of 51Z2 was estimated from the CD spectrum C using CDNN [88]. (TIF) [file pone.0062584.s002.tif]

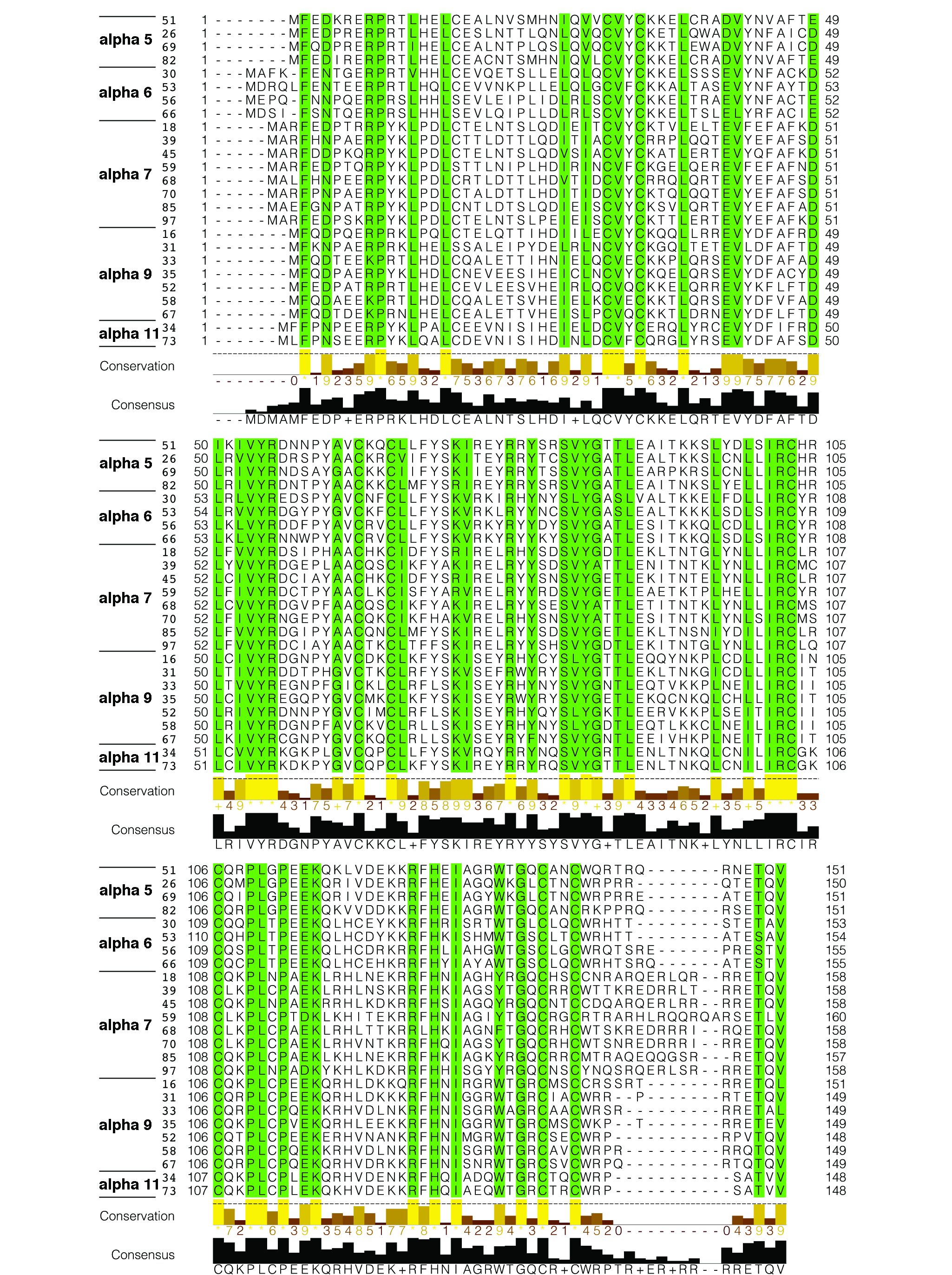

Supplement: Figure S3 — Sequence alignment of oncogenic E6 proteins. ClustalW2 [89] was utilized for alignment of the E6 proteins from oncogenic/possibly oncogenic HPV types (according to IARC, [62]). The figure was prepared using jalview [90]. Residues with a jalview-implemented conservation score [91] of 9 or higher were colored in green. The oncogenic HPV types [62] phylogenetically belong to the genus alpha-papillomaviridae and to the species indicated on the left table-side. (TIF) [file pone.0062584.s003.tif]

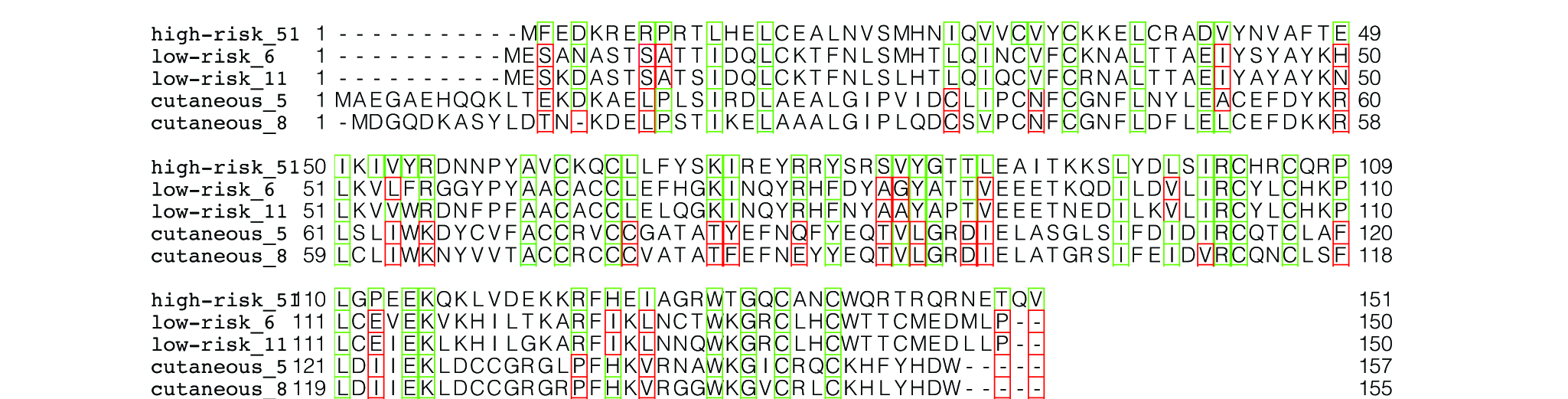

Supplement: Figure S4 — Alignment of the high-risk HPV 51 E6 to representative low-risk and cutaneous E6 proteins. The residues corresponding to conserved positions among high-risk types (Figure S3) are bracketed. The bracket color is green, if any residue present among the high-risk E6 proteins at that position is encountered (Figure S3) or red, if the residue is never observed at the respective position of any high-risk E6 protein. (TIF) [file pone.0062584.s004.tif]
