# Supplementary material for: Structural Insights into a Wildtype Domain of the Oncoprotein E6 and Its Interaction with a PDZ Domain
Source: PLoS One. 2013 Apr 30;8(4):e62584. doi: 10.1371/journal.pone.0062584 (PMC3640046; doi:10.1371/journal.pone.0062584)
Supplement: Table S1 — E6 domain architecture and solubility of recombinant E6 constructs. (PDF) [file pone.0062584.s005.pdf]

Table S1. E6 domain architecture and solubility of recombinant E6 constructs.

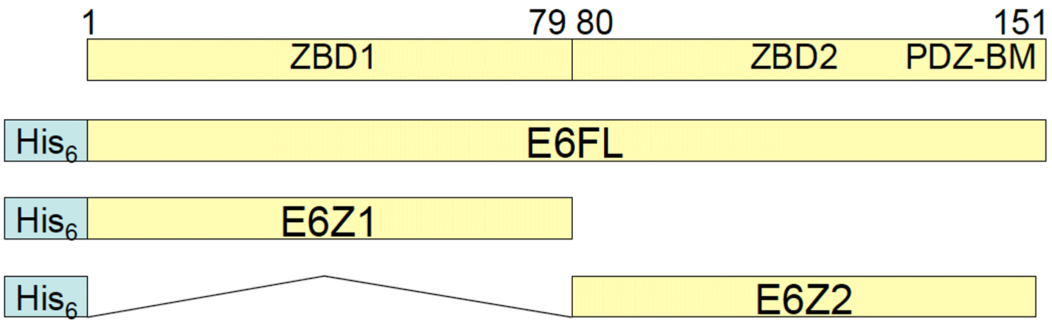

HPV type	E6FL	E6Z1	E6Z2
1a (co)	No expression 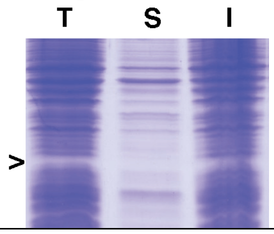	No expression 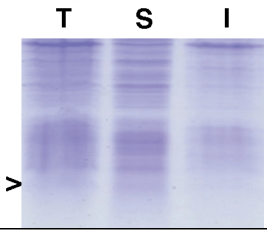	Insoluble 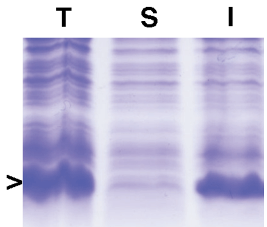
16 (co)	Insoluble 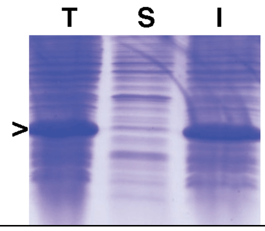	Insoluble 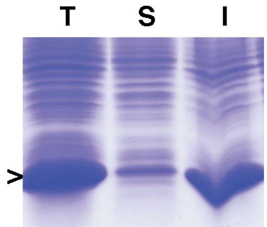	Partially soluble 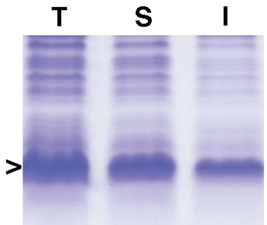
18	Insoluble 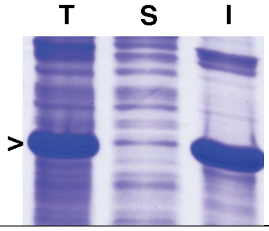	Insoluble 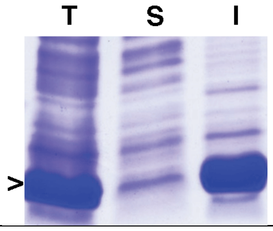	Partially soluble 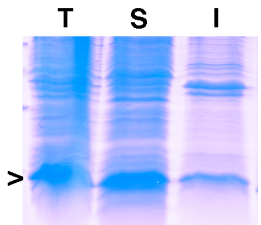
26	Insoluble 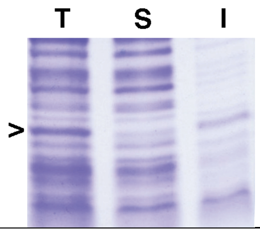	Insoluble 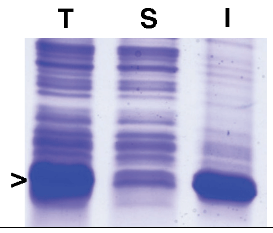	Soluble 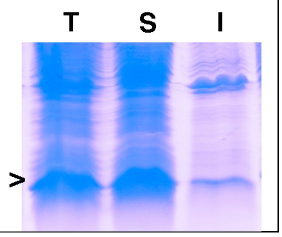

31 (co)	Insoluble<br>T S I<br>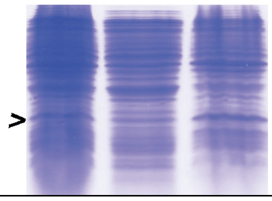	Insoluble<br>T S I<br>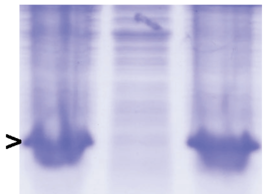	Partially soluble<br>T S I<br>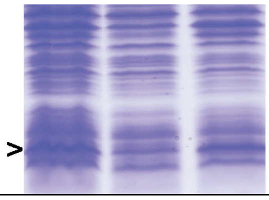
45	Insoluble<br>T S I<br>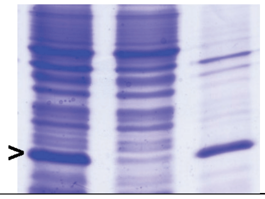	Insoluble<br>T S I<br>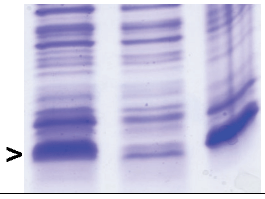	Partially soluble<br>T S I<br>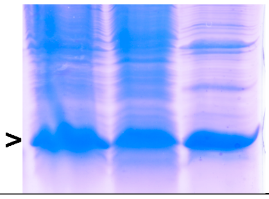
51(co)	Insoluble<br>T S I<br>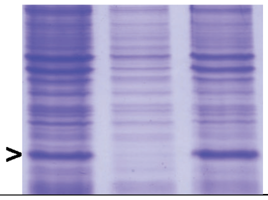	Insoluble<br>T S I<br>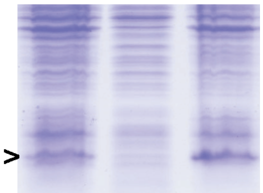	Soluble<br>T S I<br>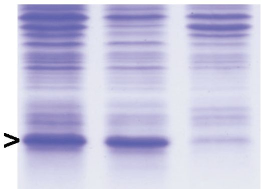
59 (co)	Insoluble<br>T S I<br>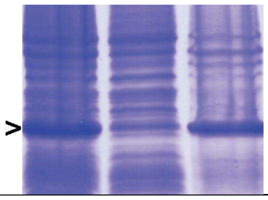	Insoluble<br>T S I<br>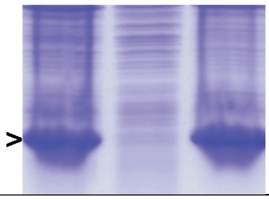	No expression<br>T S I<br>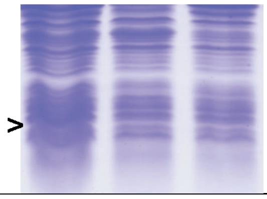
97 (co)	Insoluble<br>T S I<br>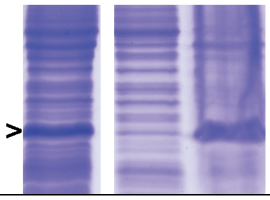	Insoluble<br>T S I<br>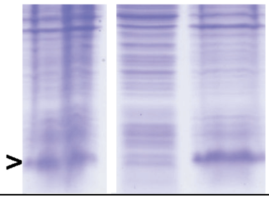	Partially soluble<br>T S I<br>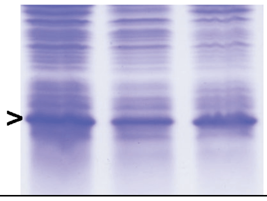

Top: E6 domain architecture. Residue numbers correspond to full-length HPV 51 E6 and for each construct zinc-binding domain (ZBD) borders were chosen by alignment to HPV 16 E6 according to [49]. Recombinant constructs were expressed in *E. coli* BL21(DE3) with an N-terminal His_6-tag linked to full-length (E6FL) or individual zinc-binding domains (E6Z1 or E6Z2), respectively. PDZ-BM denotes the PDZ-binding motif present in high-risk HPV derived E6 and missing in HPV 1a E6. Bottom: Solubility of recombinant E6 constructs in *E. coli* cell extract. Coomassie stained SDS-PAGE slices of equivalent amounts of (T) total, (S) soluble and (I) insoluble E6 containing fractions. E6 bands are indicated by (>). Codon-optimized genes for expression in *E. coli* are denoted with (co).
