# Supplementary material for: Structural Insights into a Wildtype Domain of the Oncoprotein E6 and Its Interaction with a PDZ Domain
Source: PLoS One. 2013 Apr 30;8(4):e62584. doi: 10.1371/journal.pone.0062584 (PMC3640046; doi:10.1371/journal.pone.0062584)
Supplement: Table S2 — Final screening results of E6 constructs for NMR spectroscopy. (PDF) [file pone.0062584.s006.pdf]

Table S2. Final screening results of E6 constructs for NMR spectroscopy.

Construct	Monodispersity	HSQC	Long-term stability
**51Z2**	Passed	Passed	Passed
**26Z2**	Passed	Passed	Failed
**18Z2**	Passed	Failed	
**45Z2**	Passed	Failed	
**16Z2**	Failed		
**31Z2**	Failed		
**97Z2**	Failed		

Purified, soluble E6 constructs (compare Table S1) were screened for monodispersity by gel-filtration and dynamic light scattering. Monodisperse constructs always behaved as monomers. For those constructs, [^{1}H,^{15}N]-HSQC were recorded and assessed. This test was considered as passed when the number of amino acids in the construct was matching with the number of observable amide resonances and the resonances in turn exhibited similar peak intensities. Long-term stability was considered as passed when no spectral changes occurred after two weeks post acquisition of the first [^{1}H,^{15}N]-HSQC.
