# Supplementary material for: Structural Insights into a Wildtype Domain of the Oncoprotein E6 and Its Interaction with a PDZ Domain
Source: PLoS One. 2013 Apr 30;8(4):e62584. doi: 10.1371/journal.pone.0062584 (PMC3640046; doi:10.1371/journal.pone.0062584)
Supplement: Table S3 — Details of NMR experiments and samples for the 51Z2 structure determination. (PDF) [file pone.0062584.s007.pdf]

Table S3. Details of NMR experiments and samples for the 51Z2 structure determination.

Experiment	Data points, NS (t_{mix})	Sample / Solvent
[^{1}H-^{15}N]-HSQC	2048/256, 4	1.13 mM in H_2O
HNCACB	2048/64/160, 32	1.13 mM in H_2O
CC(CO)NH	2048/96/168, 16	1.13 mM in H_2O
H(CCCO)NH	2048/64/128, 32	1.13 mM in H_2O
HNCO	2048/64/128, 8	1.13 mM in H_2O
HNHA	2048/128/96, 16	1.13 mM in H_2O
HN(CA)CO	2048/64/128, 32	1.13 mM in H_2O
[^{1}H-^{15}N]-NOESY-HSQC	2048/96/280, 16 (180 ms)	1.13 mM in H_2O
[^{1}H-^{13}C]-HSQC (ali)	1024/128, 16	1.13 mM in H_2O
[^{1}H-^{13}C]-HSQC (ali)	1024/128, 16	0.90 mM in D_2O
H(C)CH-TOCSY	2048/128/144, 8 (12 ms)	0.90 mM in D_2O
H(C)CH-COSY	2048/128/128, 16	0.90 mM in D_2O
[^{1}H-^{13}C]-NOESY-HSQC (ali)	2048/192/256, 8 (120 ms)	0.90 mM in D_2O
[^{1}H-^{13}C]-HSQC (aro)	1024/256, 4	0.90 mM in D_2O
(HB)CB(CGCD)HD	1024/128, 448	0.90 mM in D_2O
(HB)CB(CGCDCE)HE	1024/128, 512	0.90 mM in D_2O
H(CC)H-COSY aro.	2048/608, 64	1.00 mM in D_2O
[^{1}H-^{13}C]-NOESY-HSQC (aro)	2048/80/1024, 8 (120 ms)	1.00 mM in D_2O

Bruker AvanceIII NMR spectrometers with ^{1}H resonance frequencies of 750 MHz or 600 MHz were utilized. All experiments were performed with ^{13}C and ^{15}N-labeled 51Z2 and the respective implementations of pulse programs in the TOPSPIN v. 2.1 software bundle. Resolution, number of scans (NS) and, where appropriate, mixing times (t_{mix}) are given. Protein concentration was determined spectrophotometrically using a molar extinction coefficient of 13980 M^{-1} cm^{-1} as calculated from the amino acid sequence by the Protparam tool [1] at www.expasy.org/protparam/. Spectra were recorded at 283 K. 51Z2 buffer conditions were 90 mM NaCl, 45 mM L-arginine, 45 mM L-glutamate, 9 mM DTT, 0.05 % (w/v) NaN_3, pH 7.4 in 90% H_2O/10% D_2O or in 100% D_2O.
