# Supplementary material for: Structural Insights into a Wildtype Domain of the Oncoprotein E6 and Its Interaction with a PDZ Domain
Source: PLoS One. 2013 Apr 30;8(4):e62584. doi: 10.1371/journal.pone.0062584 (PMC3640046; doi:10.1371/journal.pone.0062584)
Supplement: Table S4 — Details of NMR experiments and samples for the hDlgPDZ2-E6CT11 complex structure determination. (PDF) [file pone.0062584.s008.pdf]

Table S4. Details of NMR experiments and samples for the hDlgPDZ2-E6CT11 complex structure determination.

**A** 800 µM labeled hDlgPDZ2 + 2000 µM unlabeled E6CT11		
**Experiment**	**Data points, NS (t_{mix})**	**Solvent**
[^{1}H-^{15}N]-HSQC	2048/512, 32	H_2O
HNCACB	2048/64/160, 32	H_2O
CC(CO)NH	2048/64/160, 32	H_2O
H(CCCO)NH	2048/64/160, 32	H_2O
HNCO	2048/64/128, 16	H_2O
HNHA	2048/128/48, 16	H_2O
HN(CA)CO	2048/64/128, 32	H_2O
[^{1}H-^{15}N]-NOESY-HSQC	2048/112/280, 8 (120 ms)	H_2O
[^{1}H-^{15}N]-NOESY-HSQC, f1 filtered (intermol. NOEs)	2048/64/128, 32 (120 ms)	H_2O
[^{1}H-^{13}C]-HSQC (ali)	1024/512, 32	H_2O
[^{1}H-^{13}C]-HSQC (ali)	2048/512, 32	D_2O
H(C)CH-TOCSY	2048/128/160, 8 (12 ms)	D_2O
H(C)CH-COSY	2048/128/160, 8	D_2O
[^{1}H-^{13}C]-NOESY-HSQC	2048/192/208, 8 (120 ms)	D_2O
[^{1}H-^{13}C]-NOESY-HSQC, f1 filtered (intermol. NOEs)	2048/64/128, 32 (120 ms)	D_2O
[^{1}H-^{13}C]-HSQC (aro)	2048/96, 128	D_2O
(HB)CB(CGCD)HD	1024/72, 512	D_2O
(HB)CB(CGCDCE)HE	1024/72, 512	D_2O
[^{1}H-^{13}C]-NOESY-HSQC (aro)	2048/44/288 (120 ms)	D_2O

**B** 1250 µM labeled E6CT11 + 3122 µM unlabeled hDlgPDZ2		
**Experiment**	**Data points, NS (t_{mix})**	**Solvent**
[^{1}H-^{15}N]- HSQC	2048/128, 8	H_2O
HNCACB	2048/48/118, 32	H_2O
CC(CO)NH	2048/36/152, 16	H_2O
H(CCCO)NH	2048/36/112, 16	H_2O
HNCO	2048/48/128, 16	H_2O
HNHA	2048/80/32, 32	H_2O
HN(CA)CO	2048/48/128, 32	H_2O
[^{1}H-^{15}N]-NOESY-HSQC	2048/24/160, 48 (120 ms)	H_2O
[^{1}H-^{15}N]-NOESY-HSQC, f1 filtered (intramol. NOEs)	2048/24/96, 72 (120 ms)	H_2O
[^{1}H-^{13}C]-HSQC (ali)	1024/448, 32	H_2O
[^{1}H-^{13}C]-HSQC (ali)	1024/384, 64	D_2O
H(C)CH-TOCSY	2048/128/160, 8 (12 ms)	D_2O
H(C)CH-COSY	2048/128/128, 8	D_2O
[^{1}H-^{13}C]-NOESY-HSQC	2048/128/128, 16 (120 ms)	D_2O
[^{1}H-^{13}C]-NOESY-HSQC, f1 filtered (intermol. NOEs)	2048/96/96, 40 (120 ms)	D_2O
[^{1}H-^{13}C]-NOESY-HSQC, f1 filtered (intramol. NOEs)	2048/96/96, 32 (120 ms)	D_2O

Bruker AvanceIII NMR spectrometers with ^{1}H resonance frequencies of 750 MHz or 600 MHz were utilized. All experiments were performed with ^{13}C and ^{15}N-labeled 51Z2 and the respective implementations of pulse programs in the TOPSPIN v. 2.1 software bundle. The experiments were performed with one interaction partner in ^{13}C and ^{15}N-labeled form and the other, unlabeled interaction partner in 2.5-fold excess. **A** the PDZ domain was labeled **B** the peptide was labeled. Resolution, number of scans (NS) and, where appropriate, mixing times (t_{mix}) of experiments are given. hDlgPDZ2 concentration was determined spectrophotometrically using a molar extinction coefficient of 2980 M^{-1} cm^{-1} as calculated from the amino acid sequence by the Protparam tool [1] at www.expasy.org/protparam/. Peptide concentration was determined utilizing the peptide bond absorbance [4]. Spectra were recorded at 293 K. Buffer conditions for all experiments were 20 mM sodium phosphate, 4 mM TCEP, 0.05 % (w/v) NaN_3, pH 6.5 in 90% H_2O/10% D_2O or in 100% D_2O.
