# Supplementary material for: Structural Insights into a Wildtype Domain of the Oncoprotein E6 and Its Interaction with a PDZ Domain
Source: PLoS One. 2013 Apr 30;8(4):e62584. doi: 10.1371/journal.pone.0062584 (PMC3640046; doi:10.1371/journal.pone.0062584)
Supplement: Text S1 — Methods. (PDF) [file pone.0062584.s009.pdf]

Text S1

DNA constructs for recombinant HPV E6 expression

Vectors encoding full-length E6 proteins of HPV 18, 26 and 45 were kindly provided by Matthias Dürst (Jena University Hospital). Vectors encoding codon-optimized full-length E6 reading frames flanked by NdeI/BclI restriction sites for cloning purposes and for recombinant expression in *E. coli* were obtained from DNA2.0. E6 constructs were generated as detailed in Table S1, spanning the full-length E6 protein or the amino- or carboxy-terminal part of E6, respectively.

Vectors encoding full-length codon-optimized E6 genes were transformed into chemically competent E. coli GM2163 (dam^{-}/dcm^{-}) and after amplification, DNA was isolated using the QIAGEN Plasmid Midi Kit. This DNA was digested with NdeI/BclI (New England Biolabs, NEB) following manufacturer's instructions.

All other inserts were generated by amplification of desired E6 regions by PCR. A typical PCR protocol consisted of 2 min initial denaturation, 35 cylces of 30 s denaturation (94 °C), 45 s annealing (57 °C) and 70 s elongation (72 °C) and a final elongation step for 5 min at 72 °C. 100 µl PCR reactions were carried out and contained 2 µM of each primer, 400 µM dNTPs each and 2 U *Pwo* polymerase with the corresponding polymerase buffer (PEQLAB). Forward primers for the desired protein fragment contained 5'-extensions with the NdeI cleavage site followed by E6 sequences. Reverse primers were designed to match the desired E6 region and extended to provide a stop codon followed by a BclI or BamHI cleavage site. The generated PCR fragments were run on agarose gels, excised and purified using the QIAquick Gel Extraction Kit (QIAGEN). These DNAs were then digested with NdeI and BamHI or with NdeI and BclI to obtain the desired sticky ends. The inserts were ligated with T4 DNA ligase (Life Technologies) into the pET15b vector (50 ng) predigested with NdeI and BamHI. 1 to 10 µL of each ligation mixture was transformed into *E. coli* DH5α and DNA from several colonies was isolated by QIAprep Spin Miniprep Kit (QIAGEN). Sequence identity of His_6-tagged E6 constructs was verified by sequencing (Eurofins MWG).

Expression and solubility screen of E6 constructs

Cells transformed with a vector encoding the E6 construct in question were grown to 0.7 - 0.8 OD_{600} at 37 °C with LB medium, transferred to modified M9 medium (see below) containing 10 µM Zn^{2+} and were induced after 30 min with 0.4 mM ITPG. Proteins were expressed overnight at 20 °C (~16h). Solubility was assessed after disruption of cells in suspension in lysis buffer (see below) by FRENCH® Press (for details see next section), subsequent centrifugation (10,000 x g, 4 °C, 30 min) and resuspending the pellet via ultra-sonication in a volume equal to the volume of the corresponding supernatant (see Table S1 for results).

Expression and purification of soluble E6 proteins

All E6 protein constructs cloned into pET15b were expressed in *E. coli* BL21 (DE3) using 100 mg/L ampicillin for selection. Freshly transfected cells were grown to an OD_{600} of 0.7 to 0.8 in 1L LB medium at 37 °C. Cells were harvested, washed and transferred to 250 mL modified M9 medium (6 g/L Na_2HPO_4, 4 g/L D-glucose, 3 g/L KH_2PO_4, 1 g/L $^{15}NH_4Cl$, 0.5 g/L NaCl, 2 mM $MgSO_4$, 0.1 mM $CaCl_2$, 10 µM $ZnSO_4$, 5 mg/L thiamine, 1 mg/L biotine, 1 mg/L choline cloride, 1 mg/L folic acid, 1 mg/L niacinamide, 1 mg/L D-pantothenate, 1 mg/L pyridoxal, 0.1 mg/L riboflavin). After 30 min at 37 °C, the temperature was lowered to 20 °C

and protein expression was induced with 0.4 mM IPTG. Cells were harvested by centrifugation after 16h and stored at -80 °C until further use. For purification, four to five grams of harvested bacteria were resuspended in 10 mL of lysis buffer (300 mM NaCl, 50 mM sodium phosphate, 10mM imidazole, pH 7.3, filtered, degassed) per gram (wet weight) of cells. A pinch of lyophilized RNAse A and DNAse I and one "Complete EDTA-free Protease Inhibitor" tablet (Roche) per 50 mL buffer were added. Cells were homogenized four times using a Standard FRENCH® Pressure Cell (THERMO Scientific; cell pressure: 18,000 psig). The supernatant obtained by centrifugation (30 min, 10,000 x g, 4 °C) of the lysate and containing the soluble target His_6-E6 construct was loaded two times onto a manually-packed, gravity-flow operated column with 0.5 g NiNTA agarose (QIAGEN) per gram (wet weight) bacteria pre-equilibrated with 10 column volumes (CV) of lysis buffer. Subsequently, each column was washed with lysis buffer, high-salt buffer (lysis buffer with 500 mM NaCl), and wash buffer (lysis buffer with 70 mM imidazole) until the absorbance at 280 nm reached baseline. For eluting the E6 protein constructs 0.75 CV of elution buffer (lysis buffer with 250 mM imidazole) were applied stepwise until no further protein eluted as assessed by absorbance at 280 nm. For 51Z2, typically 10 CV lysis buffer, 6 CV high-salt buffer and 16 CV wash buffer were employed before elution with a total of 6.75 CV elution buffer.

Subsequently, the pool containing the respective E6 construct was dialyzed against NMR buffer (150 mM NaCl, 50 mM L-arginine, 50 mM L-glutamine, 10 mM DTT, pH 7.4 filtered, degassed) and supplemented by thrombin (10 U per mg of E6 protein) in order to liberate E6 from the amino-terminal His_6-tag during overnight dialysis at 4 °C. The resulting E6 construct always contained the amino-terminal GSHM sequence not part of the E6 moiety but originating from the thrombin cleavage site.

Following concentration to 2 mL *via* Vivaspin 20 (3 kDa cutoff, GE healthcare), the dialyzed pool was subjected to gelfiltration using a HiLoad 16/600 Superdex 75 pg column (GE healthcare) operated at 1 mL / min NMR buffer on an ÄKTA Avant or ÄKTA Explorer FPLC system (GE Healthcare). The fractions containing the E6 protein were pooled and concentrated as above. The E6 concentration was assessed using the absorbance at 280 nm of a fresh sample with extinction coefficients calculated by the ProtParam tool [1] available at www.expasy.org/protparam/. These samples were used for biophysical assays.

Biophysical characterization of soluble E6 proteins

Analytical gelfiltration at 20 °C of E6 constructs was performed by injecting 50 µL of approx. 100 µM concentrated protein onto a TSKgelG3000SWxl column at a flow rate of 0.75 mL/min of NMR buffer. The column was calibrated at the same flow rate (see Figure S2) with thyroglobulin (669 kDa), alcohol dehydrogenase (150 kDa), carbonic anhydrase (29 kDa), cytochrome c (12.4 kDa) and aprotinine (6.5 kDa). Proteins were detected at 280 nm.

Dynamic light scattering of soluble E6 proteins (at least 100 µM in NMR buffer) was carried out on a Viscotek 802 DLS instrument. Light scattering was monitored at 90° relative to the incident laser beam. Thirty transients of 3 sec each were recorded at 20°C in a 50 µL volume. Processing of the recorded scattering intensities and the autocorrelation functions, evaluation of the mass weighted distribution of hydrodynamic radii as well as radii conversion to

molecular weights utilizing a mass model of globular proteins was carried out with the Viscotek OMNISIZE software v3.0.

$[^1H-^{15}N]$-HSQC spectra were recorded for each ^{15}N labeled E6 construct that was monodisperse as assessed by gel-filtration and dynamic light scattering. These constructs were 18Z2, 26Z2, 45Z2 and 51Z2 (Table S2). Sodium azide and D_2O was added to the E6 constructs in NMR buffer, effectively resulting in at least 100 µM E6 construct in 135 mM NaCl, 45 mM L-arginine, 45 mM L-glutamate, 9 mM DTT, 0.05 % (w/v) NaN_3, pH 7.4 in 90% H_2O/10% D_2O. Spectra were recorded at 20 °C.

Results of the biophysical characterization of soluble, purified E6 constructs are summarized in Table S2. The un-stability of 26Z2 over time is illustrated in Figure S1. The biophysical characterization of 51Z2 is given in Figure S2

Expression and purification of 51Z2

The expression was performed as described for the other soluble E6 proteins except that ^{13}C and ^{15}N labeled 51Z2 was generated for NMR spectroscopy. To that end uniformly ^{13}C-labeled D-glucose and ^{15}N-labeled NH_4Cl were used as the sole carbon and nitrogen source, respectively, in modified M9 medium. It turned out, that reducing expression time to 6 h increased yield per time unit. The purification was performed as described above except that the NaCl concentration in the SEC buffer was reduced from 150 mM to 100 mM.

Circular dichroism of 51Z2

Circular dichroism (CD) was employed to estimate the secondary structure content of 51Z2 (Figure S2c, S2d). The protein buffer was exchanged to into CD buffer (10 mM sodium phosphate, pH 7.4, filtered, degassed) via NAP 5 columns (GE healthcare). For 14.8 µM 51Z2 in a 1 mm pathlength cuvette, the CD spectrum (190-260 nm) was recorded on a JASCO spectropolarimeter J-710 using averaging of 8 scans at 20 °C. Following blank subtraction and unit conversion to molar ellipticity the resulting CD data were deconvoluted using CDNN [2].

Peptide generation

All unlabeled peptides (E6CT6: Ac-RNETQV and E6CT11: Ac-QRTRQRNETQV or pGlu-RTRQRNETQV) were chemically synthesized and kindly provided by S. Rothemund (IZKF Leipzig, Germany) in HPLC-purified form. For NMR spectroscopy of the hDlgPDZ2-E6CT11 peptide complex, ^{13}C and ^{15}N labeled E6CT11 (pGlu-RTRQRNETQV) was generated applying the intein system as previously described [3]. The concentration of peptides in solution was evaluated spectrophotometrically utilizing absorbance of peptide bonds at 205 nm [4].

Expression and purification of hDlgPDZ2

The plasmid encoding hDlgPDZ2 was obtained from addgene (www.addgene.org). hDlgPDZ2 was expressed and purified as described previously [5] utilizing the C-terminal His_6-tag for affinity chromatography followed by gelfiltration.

Surface plasmon resonance (SPR) of hDlgPDZ2

Real-time analyses were performed on a Biacore 2000 system (GE Healthcare) at 25 °C and data were processed with Scrubber 2.0c (BioLogic Software). His_6-tagged hDlgPDZ2 was immobilized on flow cell 2 of a NTA sensor chip (GE Healthcare) by using a capture coupling method that results in the capture of hDlgPDZ2 in a non-random orientation by the His_6-tag after EDC/NHS activiation [6]. 2780 RU were found to be covalently bound after injection of 1 μM hDlg-PDZ2 (50 μL) in running buffer (150 mM NaCl, 10 mM HEPES, 3 mM EDTA, 0.005% (v/v) surfactant polysorbate 20, pH 7.4) at a flow rate of 5 μL/min.

E6CT6 and E6CT11 peptides were injected in running buffer at concentrations between 3 and 200 μM. Association and dissociation times were set to 1 minute at a flow rate of 30 μL/min. Each injection was performed at least 3 times. Regeneration was achieved with 10 mM glycine/HCl, pH 2.0 for 40 seconds at a flow rate of 30 μL/min. Refractive index errors due to bulk solvent effects were corrected with responses from non-coated flow cell 1 (activated with ECD/NHS and subsequently deactivated by ethanolamine/HCl, pH 8.5) as well as subtracting blank injections. K_D values were calculated from the kinetic rate constants for peptide-hDlgPDZ2 complex formation and dissociation derived from a 1:1 interaction model including a mass transport term as well as by steady-state affinity analysis.

Comparison of hDlgPDZ2 binding to E6CT6 and E6CT11 peptides via NMR spectroscopy

[^{1}H-^{15}N]-HSQC-spectra of ^{15}N (and ^{13}C) labeled hDlgPDZ2 at 100 μM in presence of increasing concentrations (0, 20, 40, 70, 100, 200, 300 μM) of E6CT6 or E6CT11, respectively were recorded. From 200 to 300 μM peptide concentration, the signals did not show any significant further changes indicating the endpoint of titration, *i.e.* saturation of hDlgPDZ2 with peptide. In order to deduce differences of the complex of the E6CT11 versus the E6CT6 with hDlgPDZ2, the combined ^{1}H and ^{15}N chemical shifts ([7]; scaling factor ^{1}H / ^{15}N: approx. 1/5.5) obtained at the endpoints of titration (in presence of 300 μM E6CT11 or E6CT6 peptide, respectively) were compared.

NMR spectroscopy and structure calculation

NMR spectroscopy of 51Z2 in 90 mM NaCl, 45 mM L-arginine, 45 mM L-glutamate, 9 mM DTT, 0.05 % (w/v) NaN_3, pH 7.4 in 90% H_2O/10% D_2O was performed at 10 °C. For acquisition of 51Z2 NMR spectra in 100 % D_2O, an 51Z2 NMR sample (90 % H_2O, 10 % D_2O) was lyophilized and resuspended in an equal volume of 100 % D_2O. [^{1}H-^{13}C]-HSQC spectra were recorded to assess stability of 51Z2 during this procedure (data not shown).

For NMR spectroscopy of hDlgPDZ2 at 20 °C the same buffer as previously described [5] was utilized (20 mM sodium phosphate, pH 6.5, filtered, degassed, 4 mM TCEP in 90% H_2O/10% D_2O or in 100% D_2O) with the modification that 0.05% (w/v) sodium azide was included to avoid microbial growth. For NMR spectra of 51Z2 in presence of hDlgPDZ2 the 51Z2 conditions were utilized. Spectra of hDlgPDZ2 in complex with either E6CT11 or E6CT6 (51Z2-derived) peptides were recorded at the hDlgPDZ2 conditions.

^{1}H, ^{13}C and ^{15}N resonances were assigned based upon the experiments listed in Tables S3 and S4. The 51Z2 assignment, based on experiments listed in Table S3,

has a total completeness of 85.1, 88.4 and 64,0 % for ^{1}H, ^{13}C and ^{15}N atoms, respectively, with missing resonance assignments located within the first four residues (GSHM) that are vector-encoded and not part of the E6 moiety and atoms that are usually not detected by standard NMR techniques such as *e.g.* atoms within arginine guanidinium-groups or hydroxyl-protons. The assignment of 51Z2 was deposited in the BMRB, entry 18967. The assignment of the hDlgPDZ2-E6CT11 complex has been deposited in the BMRB, entry 17942 [8].

Assignments and NOEs were evaluated using CARA [9]. For structure calculation, CYANA 3.9 was employed [10,11] and in general, the best 20 of 100 structures were analyzed further. Upper limit distance constraints were derived by calibration of NOE peak intensities. For E6, calibration classes of 2.9, 3.7, 5.4 and 5.9 Å for non-exchangeable and 4.4 and 5.7 Å for exchangeable hydrogens were used, respectively. For the complexed hDlgPDZ2 calibration classes of 2.8, 3.5, 4.0, 5.0, 5.7 and 6.1 Å were employed. For the 51Z2 structure, constraints for maintenance of tetrahedral zinc coordination were included in analogy to the experimentally determined E7 zinc coordination that also carries four zinc-coordinating cysteines [12] which is consistent with the zinc coordination of HPV 16 E6 [13,14]. Titrations of 51Z2 with excess of EDTA led to severe perturbations of 51Z2 [^{1}H,^{15}N]-HSQC spectra confirming the importance of zinc for the 51Z2 structure (data not shown) and the chemical shifts of the Cα and Cβ nuclei of the 51Z2 cysteines 103, 106, 136 and 139 are consistent with their role in zinc-coordination [15]. Structural calculations were augmented by torsion angle constraints derived from TALOS+ [16] and torsion angles constraints derived from HNHA experiments [17] in combination with the FOUND module [18]. For hydrogen bonds that were consistently formed during initial rounds of structure calculation, hydrogen bond constraints were also employed in final rounds of structure calculation. Even though intermolecular hydrogen bonds were not utilized during the complex calculations, the C-terminal residues of the E6CT11 arrange as additional β-strand on the hDlgPDZ2-E6CT11 complex with the consistent formation of hydrogen bonds for these residues. Presence of *cis* prolines (ω torsion angles = 0°) was assessed by evaluating proline Cβ and Cγ chemical shifts [19]. All prolines present in 51Z2 and in the hDlgPDZ2-E6CT11 complex are in *trans* configuration relative to the preceeding residue (ω = 180°). For hDlgPDZ2 that contains eight Leu and Ile residues each, the conformation of Leu and Ile side chains was additionally evaluated: the chemical shift difference between Cδ1 and Cδ2 of Leu side chains was evaluated and the *trans* probability was calculated [20] for each methyl group. When the *trans* probability for one methyl group was above 0.75, and if a single conformation of the side chain in at least 15 out of 20 structures was observed, the χ_2 angle was constrained to the conformation mostly present in the structures (*trans*: 180°+-30°, *gauche* 60°+-30°; according to [20], such residues have a low probability of rotamer interconversions). For Ile, Cδ1 chemical shifts were evaluated to calculate the probabilities of *gauche*- and *trans* conformation [21]. When one of these probabilities was above 0.75 and at least 15/20 structures were consistent with this particular side chain conformation, the angle constraint for the corresponding χ_{21} angle was included (*gauche*-:300°+-30°, *trans*:170°+-30°).

After the final round of refinement with CYANA, the resulting best 40 out of 200 CYANA E6 and hDlgPDZ2-peptide complex structures were water-refined using RECOORD scripts [22] for CNS [23] and the 20 structures with the lowest total

energy were used for further evaluation. These have been deposited in the PDB, entries 2M3L (E6) and 2M3M (complex). Non-native residues (amino-terminal GSHM of 51Z2 and the carboxy-terminal GSHHHHHH tag of hDlgPDZ2) were not depicted in the figures of this paper and were not included in the r.m.s.d. calculations. Figures were prepared using MOLMOL [24]. PDBsum [25] providing for PROCHECK functionality was employed to evaluate torsion angle distribution of non-proline, non-glycine residues.

Superimposition of 51Z2 to other available E6 structures

The 51Z2 closest-to-mean structure of the folded core (residues 80-140) was superimposed onto the corresponding regions of HPV 16 E6 (residues 80-140; PDB ID 2LJZ) and BPV E6 in complex with the LD1 motif of paxillin (PDB ID 3PY7). When a standard sequence alignment of these proteins is generated, the BPV E6 sequence contains a supernumerary (loop) residue at position 507. Thus, for the structural alignment with identical number of atoms, for BPV E6 only residues 453-506 and 508-514 were utilized. Residue 507 is nevertheless shown in the corresponding Figure 7.

References for Supplementary Text 1

1. Gasteiger E, Hoogland C, Gattiker A, Duvard S, Wilkins MR, et al. (2005) Protein Identification and Analysis Tools on the ExPASy Server. In: Walker JM, editor. The Proteomics Protocols Handbook: Humana Press. pp. 571-607.
2. Bohm G, Muhr R, Jaenicke R (1992) Quantitative analysis of protein far UV circular dichroism spectra by neural networks. Protein Eng 5: 191-195.
3. Mischo A, Ohlenschlager O, Guhrs K-H, Gorlach M (2012) Recombinant production of isotope-labeled peptides and spontaneous cyclization of amino-terminal glutamine into pyroglutamic acid. Chembiochem 13: 1421-1423.
4. Goldfarb AR, Saidel LJ, Mosovich E (1951) The ultraviolet absorption spectra of proteins. J Biol Chem 193: 397-404.
5. Liu Y, Henry GD, Hegde RS, Baleja JD (2007) Solution structure of the hDlg/SAP97 PDZ2 domain and its mechanism of interaction with HPV-18 papillomavirus E6 protein. Biochemistry 46: 10864-10874.
6. Kimple AJ, Muller RE, Siderovski DP, Willard FS (2010) A capture coupling method for the covalent immobilization of hexahistidine tagged proteins for surface plasmon resonance. Methods Mol Biol 627: 91-9100.
7. Williamson RA, Carr MD, Frenkiel TA, Feeney J, Freedman RB (1997) Mapping the binding site for matrix metalloproteinase on the N-terminal domain of the tissue inhibitor of metalloproteinases-2 by NMR chemical shift perturbation. Biochemistry 36: 13882-13889.
8. Mischo A, Ohlenschlager O, Ramachandran R, Gorlach M (2012) NMR assignment of a PDZ domain in complex with a HPV51 E6 derived N-terminally pyroglutamic acid modified peptide. Biomol NMR Assign.
9. Keller RLJ (2004) The Computer Aided Resonance Assignment Tutorial: Cantina Verlag, Goldau.
10. Guntert P, Mumenthaler C, Wuthrich K (1997) Torsion angle dynamics for NMR structure calculation with the new program DYANA. J Mol Biol 273: 283-298.

11. Herrmann T, Guntert P, Wuthrich K (2002) Protein NMR structure determination with automated NOE assignment using the new software CANDID and the torsion angle dynamics algorithm DYANA. J Mol Biol 319: 209-227.
12. Ohlenschlager O, Seiboth T, Zengerling H, Briese L, Marchanka A, et al. (2006) Solution structure of the partially folded high-risk human papilloma virus 45 oncoprotein E7. Oncogene 25: 5953-5959.
13. Zanier K, ould M'hamed ould Sidi A, Boulade-Ladame C, Rybin V, Chappelle A, et al. (2012) Solution structure analysis of the HPV16 E6 oncoprotein reveals a self-association mechanism required for E6-mediated degradation of p53. Structure 20: 604-617.
14. Nomine Y, Masson M, Charbonnier S, Zanier K, Ristriani T, et al. (2006) Structural and functional analysis of E6 oncoprotein: insights in the molecular pathways of human papillomavirus-mediated pathogenesis. Mol Cell 21: 665-678.
15. Kornhaber GJ, Snyder D, Moseley HNB, Montelione GT (2006) Identification of zinc-ligated cysteine residues based on 13Calpha and 13Cbeta chemical shift data. J Biomol NMR 34: 259-269.
16. Shen Y, Delaglio F, Cornilescu G, Bax A (2009) TALOS+: a hybrid method for predicting protein backbone torsion angles from NMR chemical shifts. J Biomol NMR 44: 213-223.
17. Vuister GW, Bax A (1993) Quantitative J Correlation: A new Approach for Measuring Homonuclear Three-Bond J (HNHa) Coupling Constants in 15N-Enriched Proteins. J Am Chem Soc 115: 7772-7777.
18. Guntert P, Billeter M, Ohlenschlager O, Brown LR, Wuthrich K (1998) Conformational analysis of protein and nucleic acid fragments with the new grid search algorithm FOUND. J Biomol NMR 12: 543-548.
19. Shen Y, Bax A (2010) Prediction of Xaa-Pro peptide bond conformation from sequence and chemical shifts. J Biomol NMR 46: 199-204.
20. Mulder FAA (2009) Leucine side-chain conformation and dynamics in proteins from 13C NMR chemical shifts. Chembiochem 10: 1477-1479.
21. Hansen DF, Neudecker P, Vallurupalli P, Mulder FAA, Kay LE (2010) Determination of Leu side-chain conformations in excited protein states by NMR relaxation dispersion. J Am Chem Soc 132: 42-43.
22. Nederveen AJ, Doreleijers JF, Vranken W, Miller Z, Spronk CAEM, et al. (2005) RECOORD: a recalculated coordinate database of 500+ proteins from the PDB using restraints from the BioMagResBank. Proteins 59: 662-672.
23. Brunger AT, Adams PD, Clore GM, DeLano WL, Gros P, et al. (1998) Crystallography & NMR system: A new software suite for macromolecular structure determination. Acta Crystallogr D Biol Crystallogr 54: 905-921.
24. Koradi R, Billeter M, Wuthrich K (1996) MOLMOL: a program for display and analysis of macromolecular structures. J Mol Graph 14: 51-55.
25. Laskowski RA (2001) PDBsum: summaries and analyses of PDB structures. Nucleic Acids Res 29: 221-222.
